# A restatement of the natural science evidence concerning catchment-based ‘natural’ flood management in the UK

**DOI:** 10.1098/rspa.2016.0706

**Published:** 2017-03-15

**Authors:** Simon J. Dadson, Jim W. Hall, Anna Murgatroyd, Mike Acreman, Paul Bates, Keith Beven, Louise Heathwaite, Joseph Holden, Ian P. Holman, Stuart N. Lane, Enda O'Connell, Edmund Penning-Rowsell, Nick Reynard, David Sear, Colin Thorne, Rob Wilby

**Affiliations:** 1 School of Geography and the Environment, University of Oxford, South Parks Road, Oxford OX1 3QY, UK; 2 Centre for Ecology and Hydrology, Crowmarsh Gifford, Wallingford OX10 8BB, UK; 3 School of Geographical Sciences, University of Bristol, Bristol BS8 1SS, UK; 4 Lancaster Environment Centre, Lancaster University, Lancaster LA1 4YQ, UK; 5 School of Geography, University of Leeds, Leeds LS2 9JT, UK; 6 Cranfield Water Science Institute, Cranfield University, Cranfield MK43 0AL, UK; 7 Faculté des géosciences et de l'environnement, Université de Lausanne, Lausanne CH-1015, Switzerland; 8 School of Civil Engineering and Geosciences, Newcastle University, Newcastle upon Tyne, UK; 9 Flood Hazard Research Centre, Middlesex University, The Burroughs, London NW4 4BT, UK; 10 Geography and Environment, University of Southampton, Southampton, UK; 11 School of Geography, University of Nottingham, Nottingham NG7 2RD, UK; 12 Department of Geography, Loughborough University, Leicestershire, UK

**Keywords:** flood risk management, hydrology, natural flood management, science policy

## Abstract

Flooding is a very costly natural hazard in the UK and is expected to increase further under future climate change scenarios. Flood defences are commonly deployed to protect communities and property from flooding, but in recent years flood management policy has looked towards solutions that seek to mitigate flood risk at flood-prone sites through targeted interventions throughout the catchment, sometimes using techniques which involve working with natural processes. This paper describes a project to provide a succinct summary of the natural science evidence base concerning the effectiveness of catchment-based ‘natural’ flood management in the UK. The evidence summary is designed to be read by an informed but not technically specialist audience. Each evidence statement is placed into one of four categories describing the nature of the underlying information. The evidence summary forms the appendix to this paper and an annotated bibliography is provided in the electronic supplementary material.

## 1. Introduction

Flooding is among the most damaging natural hazards globally, with inundation leading to disastrous consequences including the loss of lives and destruction of property. Flooding may be fluvial, pluvial, coastal or groundwater related, or caused by a combination of these processes. Here, we focus on fluvial (river) floods, which occur when the amount of water in a river exceeds the channel's capacity. They are caused primarily by the downstream flow of run-off generated by heavy rainfall on wet ground.

Flooding is a natural process, but floodplains are also ideal for agriculture and urban development close to water resources and navigation. Consequently, development in floodplains has increased the exposure of people, property and infrastructure to floods. In many cases it is not practical, cost effective or politically feasible to relocate communities, property and economic activities away from areas prone to flooding, so measures are put in place to manage flood risk by reducing the probability of inundation and/or the negative consequences when a flood does occur.

In this restatement, we concentrate on the scientific evidence concerning the effectiveness of human interventions in river catchments that are intended to reduce fluvial flood hazard.^1^ This hazard is typically associated with high river flows. The hazard is characterized by the depth of water at locations where it may cause harm, and also by the velocity of that water, the rate of rise of water levels, duration of inundation and water quality. Interventions in river channels and floodplains that have been widely used to manage flood risk include the building of flood detention reservoirs and flood defences, channel straightening and dredging.

Recent years have seen increasing interest in management interventions that seek to modify land-use and land management, river channels, floodplains and reservoirs (where present), in order to reduce the frequency and severity of flooding, which we refer to here as ‘Catchment-Based Flood Management’ (CBFM). One subset of CBFM is ‘Natural Flood Management’ (NFM), which seeks to restore or enhance catchment processes that have been affected by human intervention. These activities aim to reduce flood hazard, while also sustaining or enhancing other potentially significant co-benefits including enhanced ecosystem services (aquatic, riparian and terrestrial) such as greater biodiversity, improved soil and water quality, carbon sequestration, reduced soil erosion, greater agricultural productivity and improved public health and well-being.

While it is recognized that implementation of CBFM or NFM can produce multiple co-benefits, it is not easy to establish the precise nature and extent of those benefits. Often a complex set of trade-offs exists between costs and benefits that accrue to different stakeholder groups within and outside the catchment. Also, while the benefits are well understood in principle, uncertainty around the quantitative predictions of the potential for CBFM/NFM interventions to reduce local and downstream flood hazards remains high, especially in large catchments and for major floods. Differences between river catchments make it difficult to transfer empirical evidence from one location to another. The relative importance of the multiple factors that influence flooding varies spatially and with time, which means that even if an intervention may be beneficial locally, a positive impact on flooding downstream cannot be guaranteed for all possible events in all locations.

The aim of this restatement is to review the scientific evidence for the impacts of CBFM and NFM strategies on downstream flood hazard in the UK. Here, we focus on the natural science evidence base; the social sciences and economics also provide important evidence for policy-making but this is not considered here. The objective is to review processes that impact flood frequency^2^ and hazard potential, principally with respect to flood volumes and flood levels but also velocity, duration and water depth. These include modifications to land cover and land management to retain water on and within the land before it flows into rivers, and modifications to and protection of channels and rivers to slow the flow of water and reduce water levels in floodplains downstream where there is a flood hazard (table 1).

**Table 1.** Catchment-based measures that could contribute to flood management. After [1].

Flood Risk Management theme	specific measure	examples
retaining water in the landscape: water retention through management of infiltration and overland flow	land-use changes	arable to grassland conversion, forestry and woodland planting, restrictions on hillslope cropping (e.g. silage maize), moorland and peatland restoration
	arable land-use practices	spring cropping versus winter cropping, cover crops, extensification, set aside
	livestock land practices	lower stocking rates, restriction of the grazing season
	tillage practices	conservation tillage, contour/cross slope ploughing
	field drainage (to increase storage)	deep cultivations and drainage to reduce impermeability
	buffer strips and buffer zones	contour grass strips, hedges, shelter belts, bunds, riparian buffer strips, controls on bank erosion
	machinery management	low ground pressures, avoiding wet conditions
	urban land-use	increased permeable areas and surface storage
retaining water in the landscape: managing connectivity and conveyance	management of hillslope connectivity	blockage of farm ditches and moorland grips
	buffer strips and buffering zones to reduce connectivity	contour grass strips, hedges, shelter belts, bunds, field margins, riparian buffer strips
	channel maintenance	modifications to maintenance of farm ditches
	drainage and pumping operations	modifications to drainage and pumping regimes
	field and farm structures	modifications to gates, yards, tracks and culverts
	on-farm retention	retention ponds and ditches
	river restoration	restoration of river profile and cross-sections, channel realignment and changes to planform pattern
	upland water retention	farm ponds, ditches, wetlands
making space for water: floodplain conveyance and storage	water storage areas	on- or off-line storage, washlands, polders, impoundment reservoirs
	wetlands	wetland creation, engineered storage scrapes, controlled water levels
	river restoration/retraining	river re-profiling, channel works, riparian works
	river and water course management	vegetation clearance, channel maintenance and riparian works
	floodplain restoration	setback of embankments, reconnecting rivers and floodplains

## 2. Material and methods

The restatement is intended to provide a succinct summary of the natural science evidence relevant to policy-making in the UK as of June 2016. The restatement offers a consensus judgement on the strength of the different evidence components using the abbreviated codes established in previous Oxford Martin School restatements:

[D_ata_] a strong evidence base involving experimental studies or field data collection, with appropriate detailed statistical or other quantitative analysis;
[E_xp_op_] a consensus of expert opinion extrapolating results from relevant studies and well-established principles;
[S_upp_ev_] some supporting evidence but further work would improve the evidence base substantially; and
[P_rojns_] projections made using well-established models that are based on the available physical principles and/or robust empirical evidence gathered in a wide range of settings.

The categories employed are based on those used in previous restatements [2,3], which were themselves developed from the medical and climate change literature. The statements are qualitative in nature and are not intended to form a ranking. We note that, in many cases, evidence is context- or scale-specific. Moreover, interventions that may be effective in one location and at one scale may have a different effect in another setting. Where further gradation is necessary to reflect the quality of evidence, this is done in the accompanying text.

We note in particular the wide range of models used in hydrological science. Some models are based on well-established physical principles such as conservation of mass, energy and momentum, which are fundamental properties of physical systems but which nonetheless require generalizations about parameter values or model equations in order to be applied. Other models represent generalizations from necessarily limited sets of observations whose conclusions cannot be expected to hold in settings different from those in which they were generated.

## 3. Results

The summary of the natural science evidence base relevant to catchment-based ‘natural’ flood management in the UK is given in the appendix, with an annotated bibliography provided as the electronic supplementary material.

## 4. Discussion

In this restatement, we have drawn attention to some important evidence gaps. We highlight several immediate priorities:

1. National monitoring networks provide essential data for estimating flood risk and determining the efficacy of interventions. Maintenance and enhancement of monitoring systems should pay particular attention to the accurate measurement of high water levels and out-of-bank flows. Significant uncertainty about the impacts of different types of intervention both when used individually and in combination arises in part because there has not been sufficient research to establish causal links between CBFM and NFM actions and downstream effects. Long-term monitoring is necessary because major floods are rare events; it is also necessary that prospective studies establish good experimental controls and collect accurate baseline data.
2. Recent model studies have begun to reproduce field measurements from relatively small monitored catchments. These models could now be used to simulate the impact of changes in land use and management practices in larger catchments. Model studies of large recently flooded catchments (e.g. Yorkshire Ouse, Eden, Parrett, Thames) could help to establish the scale and spatial location of different types of catchment-based intervention that might be required to have a notable effect on flooding. It is important to investigate whether the models' findings can be extrapolated to regions larger than those for which they have been evaluated, given the constraints posed by their formulation and uncertainties in validation data, and to understand whether the benefits of CBFM/NFM measures are more, less or equally predictable than the benefits of hard engineered assets.
3. The Environment Agency's Catchment Flood Management Plans (CFMPs) assess flood risks across a catchment and can include maintaining or restoring natural processes among the measures that might be taken in the course of flood risk management. Moreover, a large number of catchment-based schemes are currently underway, promoted by Rivers Trusts, Wildlife Trusts and flooded community groups, for example. Many of these local initiatives are neither being planned nor evaluated at larger spatial scales. The lack of monitored baselines and experimental controls creates a risk that the wider and scale-dependent impacts cannot be properly investigated or used to inform decision-making. Research and data available within the water management industry (e.g. water companies, Internal Drainage Boards, land and estate management organizations) would add to the evidence base if it were disseminated more widely.
4. The performance, longevity and operation and maintenance of CBFM/NFM should be systematically compared with traditional engineering solutions. The risks and uncertainties and benefits associated with each approach need to be more fully understood and communicated. The interactions between fluvial floods and other flood types (e.g. pluvial, coastal and groundwater) and sequences of events also warrant further systematic study. The potential for CBFM/NFM interventions in groundwater-dominated and heavily engineered and drained river systems needs further research. The extent to which these interventions add resilience to the impacts of climate change is also worthy of further investigation.
5. A practitioner toolkit would help to share practical experience (while noting context-specific issues), paying attention to appropriate design criteria. A practitioner toolkit might comprise a set of documents outlining best practices and the situations in which their effectiveness has been demonstrated, drawing on well-studied examples. This could be accompanied by a protocol for coordinated, high quality, monitoring of the catchment, river corridor and hydro-meteorological conditions, drawing on modern sensor, communications and information technologies.
6. There would be benefits from improved communication and collaboration between groups undertaking research in river catchments (e.g. water quality, sediment transport, river restoration, biodiversity, agriculture and forestry), which are all relevant to flood risk management. On the basis of current evidence, the cost-effectiveness of NFM at medium-large scales is likely to rely on understanding interactions between flows, debris and sediment management taking into account the range of ecosystem service benefits that accompany NFM.

## Supplementary Material

Annotated Bibliography

## Supplementary Material

Table 2

## Figures and Tables

**Figure 1.**
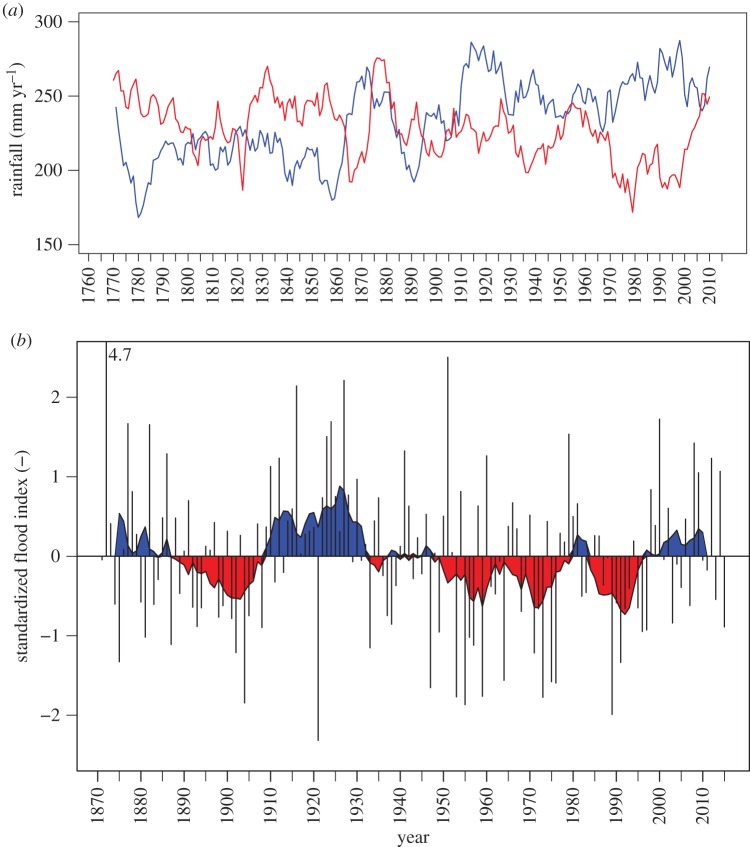
Climate variability and flooding. (*a*) England and Wales precipitation seasonality (1776–2015); the blue line shows winter (DJF) precipitation; the red line indicates summer (JJA) precipitation. Data from [4]; http://www.metoffice.gov.uk/hadobs/hadukp/. (*b*) Annual mean flood index (1871–2015). The blue and red shading shows flood-rich and flood-poor periods respectively Data from ref. [5]; https://crudata.uea.ac.uk/cru/data/lwt/.

**Figure 2.**
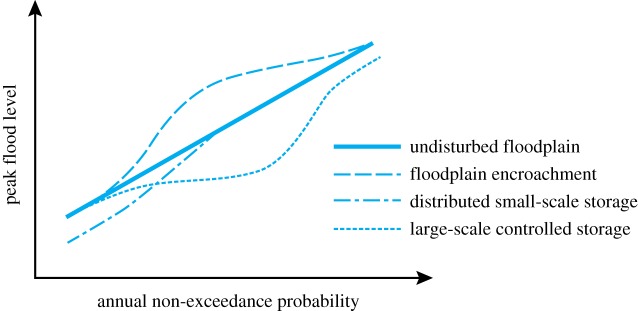
Hypothesized impact of three types of engineering activities on the flood frequency curve. After [6].

**Figure 3.**
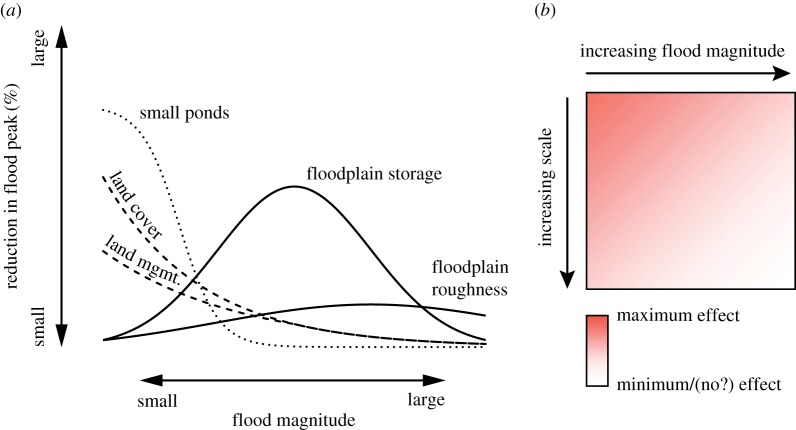
Schematic diagram showing relative effects of catchment-scale interventions on flood peaks. (*a*) Effect of different types of intervention on flood peak reduction [E_xp_op_]; (*b*) combined effect of CBFM interventions with flood magnitude and catchment scale [E_xp_op_]. We note that the effects achievable in practice will depend on the details of the particular intervention and the context in which it is deployed.

## Footnotes

1 It is conventional to distinguish between the three components that constitute flood risk to people and economies: *hazard* is the phenomenon with potential to cause harm (i.e. unusually high water levels); *exposure* describes the people or assets in harm's way; and *vulnerability* is the susceptibility of people and property to loss when exposed to a hazard.

2 Flood frequency is a measure of likelihood, which in this restatement we measure using Annual Exceedence Probability (AEP). The AEP is the chance of a flood of this magnitude or greater occurring in any particular year.

## Appendix A. A restatement of the natural science evidence concerning catchment-based ‘natural’ flood management in the UK

**(a) Introduction and aims**

(1) *Background*

Flooding causes hundreds of millions of pounds of damage in the UK and climate change is projected to increase the frequency and intensity of heavy rainfall in the future. Flooding may be fluvial, pluvial,^3^ coastal or groundwater related, or a combination of these. Here we focus on fluvial floods, which are primarily caused by the downstream flow of run-off generated by heavy rainfall on wet ground. Most investment to reduce flood risk goes into engineered systems like flood defences and channel modification. ‘Catchment-Based Flood Management’ (CBFM) consists of interventions of any kind that seek to modify land-use, land management, upstream river channels and floodplains, in order to reduce the frequency and severity of flooding. CBFM aims to alter flood risk by making changes within the wider catchment rather than managing flood hazard locally at the point where flooding occurs. One subset of CBFM is ‘Natural Flood Management’ (NFM^4^) which seeks to restore catchment and river processes that have been adversely affected by human intervention. CBFM and NFM may help reduce the frequency and severity of flooding as well as delivering other environmental, social and economic benefits. However, because CBFM and NFM interventions often occur alongside other factors that influence flooding, including spatial and temporal variability in rainfall and run-off, assessing the effectiveness of these interventions is challenging.

(2) *Principles*

The magnitude of fluvial flooding depends upon (i) the rate of run-off from hillslopes into river channels, (ii) the rate of propagation of the run-off downstream in river channels, and (iii) how run-off contributions from multiple hillslopes and sub-catchments combine via the channel network to generate the downstream flood hydrograph. In small catchments,^5^ the peak of the flood hydrograph is dominated by run-off from hillslopes in response to storm rainfall. In larger catchments, the river channel network determines which areas of the catchment contribute to the peak of the flood hydrograph to cause flooding [D_ata_]. The impact of NFM/CBFM measures on flooding therefore depends on their location within the catchment, the size of the catchment and the connectivity of the channel network [P_rojns_]. Simple extrapolation of small-scale changes to larger catchment areas is therefore not possible, and the effects of NFM/CBFM must be assessed within the context of the whole catchment [E_xp_op_]. Relatively few studies adopt such a catchment-scale framework; some of those that do are summarized in §20 [E_xp_op_].

(3) *Evidence*

Many individual studies have investigated the direct effect on run-off and river flow of variations in natural land cover, human-modified land-use and specific details of land and river channel management practices. Several integrated studies have investigated the potential effect of CBFM and NFM on flooding. Together, these strands of research have generated a large amount of important, policy-relevant information. However, because each flood is a consequence of a unique combination of conditions, evidence needs to be interpreted with care. Here, we summarize the evidence base relevant to policy-making in the areas of CBFM and NFM, in the UK, as of June 2016. We look principally at evidence from the UK, but make reference to studies undertaken overseas where appropriate. We focus mainly on peer-reviewed academic studies, although we have indicated the existence of practitioner-led evidence databases and catalogues where relevant.

(4) *Aim*

We provide a consensus judgement on the nature of the different evidence components using the abbreviated codes, which are based on those used in previous Oxford Martin School Restatements:

[D_ata_] a strong evidence base involving experimental studies or field data collection, with appropriate detailed statistical or other quantitative analysis;
[E_xp_op_] a consensus of expert opinion extrapolating results from relevant studies and well-established principles;
[S_upp_ev_] some supporting evidence but further work would improve the evidence base substantially; and
[P_rojns_] projections made using well-established models that are based on the available physical principles and/or robust empirical evidence gathered in a wide range of settings.

**(b) Meteorological drivers of flooding**

(5) *Meteorological data and trends*

The UK benefits from a meteorological observation network that is dense by global standards. Annual precipitation totals vary considerably from year to year, but there has been no detectable long-term change in spatially averaged annual precipitation totals since the eighteenth century [D_ata_]. Over this time period, the UK has, however, experienced a statistically significant increase in winter precipitation, and a reduction in summer precipitation (figure 1*a*) [D_ata_]. Winter precipitation in uplands has increased more than in lowlands [D_ata_].

(6) *River flow variability and trends*

Extensive catchment and river channel modifications including impoundments, diversions and water withdrawals have modified river flow, making climate-driven trends, if present, difficult to detect. The challenge is exacerbated by changes in measurement techniques and instrument locations. ‘Benchmark’ river basins that have not experienced widespread channel modification, abstraction or urbanization during the period of record show a pattern of increased winter extremes between the 1960s and the early-2000s in the north and west of the UK, but this trend is not present in their southeastern counterparts [D_ata_].

(7) *Flood magnitude and frequency*

Increases in flood frequency do not always imply increases in flood magnitude [D_ata_]. The longest UK river flow datasets show that flood magnitudes observed since 1960 are not unusual compared with earlier observations. The Thames, which has the longest gauged record in the UK, shows no significant long-term trend in flood magnitude since 1883 [D_ata_]. Similar results hold for the Wye and Scottish Dee, back to the 1930s [D_ata_].

(8) *Flood-rich and flood-poor periods*

Climate variability results in clusters of flood-rich (e.g. 1908–1934, and 1998–present) and flood-poor periods (e.g. 1950–1980; figure 1*b*) [D_ata_]. Flood-rich episodes are associated with westerly airflows and cyclonic conditions across the UK [D_ata_]. The flood-rich period starting in the late-1990s has been attributed to warmer conditions in the North Atlantic Ocean [S_upp_ev_]. Sedimentary deposits laid down after torrential flood flows in small catchments provide evidence for a similarly flood-rich period between 1840 and 1890; in the seventeenth to nineteenth centuries floods were both more frequent and more severe than those experienced since 1998 [D_ata_].

(9) *Climate change projections*

Projections from the latest global and regional climate models do not suggest a systematic change in annual rainfall totals in the UK between now and 2080 (80% of the simulations show between a 16% reduction and a 14% increase) [P_rojns_]. The models suggest some change in the spatial distribution of rainfall, with a projected increase in winter rainfall on the west coast of between +9% and +70%, and reduction in summer precipitation in southern England of −65% to −6% by 2080 [P_rojns_]. Higher rainfall maxima are expected, and storms are expected to occur more often, especially in the summer [P_rojns_]. Winter upland rainfall totals may also increase [P_rojns_]. Under warmer conditions, winter precipitation is more likely to be in the form of rain rather than snow [P_rojns_].

**(c) The effects of land cover and land management on flooding**

(10) *Historical changes in land cover*

Land cover has changed radically in the UK due to human influence, with forest covering much of the UK in prehistoric times and declining to a minimum of 6% in 1930 and then increasing to 12% currently (2007 figures) [D_ata_]. There have been major changes to agricultural practices, upland management, and to the extent and type of urbanization [D_ata_].

(11) *The influence of land cover on flooding*

At small spatial scales (less than 20 km^2^) the effect of land cover and land management on flood flows is evident in some studies, but not for the most extreme floods [S_upp_ev_]. Measured data for land-use impacts in larger catchments (more than 100 km^2^) are lacking [S_upp_ev_]. The Flood Studies Report and Flood Estimation Handbook concluded (from studies of 553 and 943 catchments, respectively) that urban extent was the only land cover factor that was significantly related to the magnitude of the mean annual flood in UK rivers [D_ata_]. Numerical modelling suggests that the effect of land cover changes on river flows in the Thames catchment is small compared with natural climatic variability [P_rojns_].

(12) *Effects of forest cover*

The impacts of upland conifer forestry on water availability and run-off have been the subject of several experimental studies in the UK. One of the longest-running investigations was based in two UK experimental catchments at Plynlimon (10.6 km^2^ and 8.7 km^2^) in mid-Wales where:

(a) Mature forest produced higher evaporative losses than grassland under equivalent conditions, owing to the greater amount of water intercepted within the tree canopy [D_ata_].
(b) For smaller storms (less than 20% of the mean annual flood), flow peaks per unit area were smaller in the forested catchment than under grassland although it is noted that these storms do not usually pose a significant flood hazard [D_ata_].
(c) By contrast, during high flood flows no significant difference was found between flood peaks (per unit area) in the two Plynlimon catchments [D_ata_].

Limited suppression of flood peaks in the forested catchment was attributed to the relatively small amount of canopy storage and generally drier soils beneath forest stands compared with grassland [E_xp_op_]. Under sustained winter rainfall, soil saturation will occur and little mitigation of high flood flows would be expected [E_xp_op_].

(13) *Timber planting and harvesting; forestry operations*

Planting forests and harvesting timber can have long-lasting effects on run-off, stream flow and flood risk due to soil compaction by machinery, construction and use of forest roads, artificial drainage and by increasing soil loss. The magnitude of these effects depends critically on management practices [D_ata_].

(a) Evidence from Europe and North America shows timber harvesting can exacerbate peak flows and lead to flooding [D_ata_]. UK studies (e.g. Plynlimon) show augmented low flows but not increased peak flows [D_ata_]; the difference with international studies is attributed to good forestry practice [E_xp_op_].
(b) In two small highland catchments in Scotland, approximately 30 km northeast of Stirling at Balquhidder (6.85 and 7.70 km^2^), clear-felling of 50% of the catchment is estimated (with the aid of a model) to have led to a small increase in total flow of approximately 3%. The calculated difference is likely to be within the range of model calibration uncertainty [P_rojns_].
(c) Establishment of conifer plantations in the 1.5 km^2^ Coalburn catchment in the Kielder Forest (northwest England) on previously rough grazing land increased the rate of run-off after storms for 20 years [D_ata_], probably due to improvements to drainage prior to planting [E_xp_op_]. Once the plantation forest in this location reached maturity, a 200–300 mm decrease in annual run-off was observed, compared with that prior to afforestation [D_ata_].
(d) Simulated peak flows are higher at Coalburn when small trees are present compared with taller trees, but the bigger the flood the smaller is the difference [P_rojns_].

(14) *Impact of agricultural practices*

Changing agricultural practices over the last century have led to (i) removal of hedgerows to create larger fields; (ii) soil compaction; (iii) land drains; (iv) increased flow through cracks and sub-surface drains (macropore flow); (v) concentrated overland flow in ditches, tracks and wheel tracks; (vi) narrowing or removal of non-agricultural riparian corridors and buffer strips; and (vii) changes in crop type and switches from spring-sown to autumn-sown arable crops. Grassland management practices, including intensive grazing, have led to soil structural degradation in local cases and these changes have been shown to increase run-off production [D_ata_]. Localized increases in flooding at the plot and hillslope scale have been attributed to changes in land cover, crop type (including the expansion of crops such as maize) and intensification of farming [D_ata_].

(15) *Impact of land drainage*

Drainage to control water levels has complex effects on run-off, which depend on the type of drainage used and the sequence of events.

(a) At the plot scale, drainage reduces peak flows from impermeable (e.g. clay) soils, but increases those from more permeable soils [D_ata_].
(b) By drying soils, drainage increases their capacity to store water after a rainfall event, but when the soils become saturated drainage increases flows [D_ata_].
(c) At the plot scale, higher flow peaks result from open ditches compared with sub-surface drains. Higher peaks arise from the use of a ‘mole plough’ to create sub-surface channels in impermeable soils (‘mole’ drainage) in combination with other forms of sub-surface drainage [D_ata_].
(d) Extension of drainage networks up hillsides increases the speed with which run-off is transported to rivers [D_ata_].

(16) *Soil compaction*

Both arable and livestock agriculture practices can cause surface and sub-surface soil compaction, and at local scales this has been demonstrated to increase surface run-off [D_ata_]. Effects at catchment scale have not been identified, though there have been few relevant studies [E_xp_op_].

(a) Soil compaction due to higher livestock density increased the flood peak after a storm in northwest England by 7%, according to a model simulation in the 36 km^2^ Scandal Beck tributary of the River Eden in northwest England [P_rojns_].
(b) A recent survey in southwest England has shown that 75% of survey plots that had been planted with late-harvested crops (e.g. maize or potatoes) suffered severe degradation of soil structure due to soil compaction, generating additional surface run-off and surface water pollution, and reducing aquifer recharge [D_ata_].
(c) In a plot-scale experiment at Pontbren in mid-Wales, tree-planted plots produced between 48% and 78% less run-off than grazed control plots, although there was a high degree of variability between sites [D_ata_]. Five years on, soil infiltration rates were 67 times higher in tree-planted plots compared with grazed pasture, and the effect of tree planting was separate from the effect of excluding sheep [D_ata_].

(17) *Upland and peatland impacts*

Upland areas often receive heavy rainfall. Management practices affect peak water flows downstream [D_ata_]. Most evidence demonstrating flood response to upland interventions is at the small catchment scale (less than 20 km^2^) rather than at the large catchment scale (more than 100 km^2^).

(a) Higher flood peaks and shorter times to reach peak flow are associated with peatland degradation and removal of vegetation cover [D_ata_].
(b) Locating dense ground cover such as *Sphagnum* moss along more gentle gradient slopes and near watercourses has the greatest impact on flood peak reduction (or the converse for bare ground) when compared with having the same proportion of dense surface cover elsewhere in the catchment [P_rojns_].
(c) A study of the effects of heather burning to encourage grouse found mixed effects: slower run-off after moderate rainfall (owing to deeper water tables) but faster run-off for the highest 20% of events (owing to faster flow over sparsely vegetated, saturated ground) [S_upp_ev_].

(18) *Upland ditch blocking*

Upland ditches may increase or decrease flood peaks at the local scale depending on the layout of the drains, topography and flood peak synchronization in the main channel [P_rojns_]. Upland ditch blocking (i.e. using sequences of dams along each ditch) has been common over the last 15 years in the UK, mainly to benefit biodiversity or water quality or both.

(a) Ditch blocking in upland peat is effective in reducing flow peaks only in the steepest, smoothest drains; surface roughness of the surrounding vegetated peat may be more important than the presence or absence of ditches [P_rojns_].
(b) A 5-year monitoring study in the Peak District showed that the effects of drains depended on their configuration, and on the velocity differential between overland flow and flow in the drains themselves [D_ata_]. Modelling studies have suggested that ditch blocking can sometimes reduce peak flows, though to a degree dependent on the details of topography and how ditches are blocked [P_rojns_].

(19) *Impacts of urbanization and sustainable drainage systems (SuDS)*

Urbanization tends to increase peak flood flows because of reduced infiltration under paved areas and rapid flow over the surface, along channelized streams and through culverts and pipes [D_ata_]. Urban flooding is generally greatest from intense convective storms in summer [D_ata_].

(a) Engineering interventions, including permeable paving, stormwater retention and storage basins and sustainable drainage systems (SuDS), can avoid, mitigate or even reverse the adverse effects of urbanization on surface run-off [D_ata_];
(b) Restoration of urban watercourses and their vegetated riparian corridors, plus reconnection of their floodplains can be used to convey or store urban run-off while encouraging infiltration and improving water quality [D_ata_].

(20) *Catchment-scale effects of land management practices*

Understanding how local changes in land cover and land management affect water flows and flood risk downstream in large catchments is a major research challenge, which has been addressed by several large projects including the Catchment Hydrology and Sustainable Management Programme (CHASM), Flood Risk Management Research Consortium (FRMRC) and Flood Risk from Extreme Events (FREE) research programmes. Nonetheless, the hydrological responses to land-use change tend to be context-specific and translating results between one context and another is difficult.

(a) Under the United Utilities' Sustainable Catchment Management Plan (SCaMP) project, changes to upland land-use management have been carried out in the 260 km^2^ Hodder catchment (a tributary of the River Ribble, northwest England) primarily to reduce suspended sediment and coloration in water used for public supply. The changes, which covered 25 km^2^ within a 58 km^2^ sub-catchment, included moorland ditch blocking in areas of blanket peat, tree planting and reduction in livestock stocking density. The possible consequences of these changes for downstream flooding were evaluated at multiple scales, to test whether small-scale impacts propagate through the river network. SCaMP changes had minimal short-term effects on the pattern of flood flows [D_ata_]. No effects were found at the larger scale of the entire Hodder catchment (260 km^2^) during the period of study [D_ata_]. This finding was corroborated by a modelling study that showed that the median reduction in the flood peak associated with an extreme rainfall event produced by a realistic suite of land-use management changes was only 2%, assuming that channel conveyance did not change, but with an uncertainty range of a 1% increase to a 6% decrease [P_rojns_].
(b) Results from the Pontbren multi-scale experiment illustrate the potential use of tree shelterbelts to reduce plot-scale run-off [E_xp_op_]. Plot-scale monitoring took place on 12 × 12 m plots at four sites in a 12 km^2^ catchment in the headwaters of the Upper Severn in mid-Wales (see §16). Field-scale monitoring and modelling were used to investigate the impacts of tree shelterbelts. Small catchment-scale monitoring looked at how land-use impacts flows and sediments (see §22).
  i. A field-scale modelling study suggested that planting tree shelterbelts near the bottom of all improved grassland fields in a 6 km^2^ sub-catchment might reduce peak flows by 13–48% for the largest storm seen in the study period (peak rainfall intensity 54 mm h^−1^), with a 15 min reduction in time-to-peak [P_rojns_].
  ii. For a hypothetical extreme storm with rainfall of 140 mm over 2 days (estimated 0.6% Annual Exceedance Probability (AEP)^6^) the simulated reduction in peak flows was 2–11% and there was no reduction in time-to-peak [P_rojns_].
  iii. The authors note the high levels of uncertainty associated with their model predictions and leave open the question of whether reductions in flood peaks would be possible at spatial scales larger than 6 km^2^ [E_xp_op_].
(c) In the River Axe catchment (288.5 km^2^) in southwest England, an assessment of the observable historical effects of land-use change on basin-scale run-off showed they are limited to high flows that arise from moderate rainfalls (10–30 mm day^−1^) after a period of dry weather [D_ata_]. For flows of this magnitude (which usually remain within the river's channel), the results suggest that farming practices that minimize soil degradation and compaction may produce a reduction in river flows in this catchment [S_upp_ev_]. The authors note, however, that in nine other catchments no significant changes could be identified, owing to natural variability and data limitations [D_ata_].

(21) *Summary*

There is clear evidence that appropriately chosen land-use and land-cover interventions can reduce local peak water flows after moderate rainfall events [D_ata_]. The evidence does not suggest these interventions will have a major effect on nearby downstream flood risk for the most extreme events [S_upp_ev_]. The evidence available for the downstream effects of upstream land-use changes at large catchment scales is more limited, but at present it does not suggest that realistic land-use changes will make a major difference to downstream flood risk [E_xp_op_]. Moreover, it should be recognized that, although the UK landscape has undergone extensive change due to the multiplicity of intensive farming interventions over many decades, the effects of these interventions on flooding have been difficult to detect. Long-term monitoring is needed to separate the effects of land management from those of climatic variability; without this it is unwise to extrapolate the findings from individual studies to larger scales, or to settings with different soil and vegetation types [E_xp_op_].

**(d) Channel flow**

(22) *Geomorphic processes and river channel form*

Erosion, transport and deposition of sediment can, over time, result in major changes in channel morphology (cross-section and profile) and even channel pattern in some cases [D_ata_]. If the amount of sediment flow from a catchment increases, some of it will tend to accumulate downstream in places where the pattern of water flow is insufficient to keep material in suspension. Sedimentation reduces the channel's capacity to convey flow, resulting in higher water levels for a given discharge, and increasing the frequency of flooding [D_ata_]. These processes are commonly overlooked in flood risk mapping exercises, but are likely to be important in any river system which receives high rates of sediment delivery and which in the past would have deposited much of its sediment on the floodplain [E_xp_op_].

(a) A simulation study of a river in Yorkshire showed that over a 16-month period change in channel configuration due to coarse sediment deposition led to a substantial increase in the area that flooded for the highest flows that were recorded during the study period [P_rojns_].
(b) In a simulation study of 41 rivers across England and Wales it has been calculated that, on average, a 10% reduction in channel capacity would increase flooding by 1.5 days per year and that, conversely, a 10% increase in channel capacity would decrease flooding by 1.5 days per year [P_rojns_].
(c) Some agricultural land practices are known to cause sediments to accumulate in drainage channels and rivers [E_xp_op_]. Annual surveys of sediment accumulation in 10 small wetlands built on four farms in Cumbria and Leicestershire have shown that on-farm interventions can trap significant quantities of sediment (in this case 0.04–0.8 t ha^−1^ y^−1^), particularly during intense rainfall at times when crop cover is poor [D_ata_].
(d) In Pontbren in mid-Wales (see §20), sediment loads were 5–12 times higher in a stream draining improved grassland than in one draining traditionally managed moorland, with implications for sediment delivery downstream [D_ata_].

(23) *Hydraulic effects of channel modification*

Traditional flood-control channel designs have included enlarging the natural channel cross-section and straightening meanders to increase the hydraulic gradient and therefore conveyance of water. While increasing the channel cross-section will reduce local water levels, the resulting higher flows can increase the flood hazard downstream [E_xp_op_]. In a large flood, when much of the water flow is outside the river, the effect of channel modification is relatively small [E_xp_op_].

(24) *Sedimentary effects of channel modification*

Excessive widening or deepening of natural watercourses can initiate channel instabilities resulting in erosion and sedimentation, requiring maintenance work to preserve the design capacity of the scheme [E_xp_op_]. Dredging to re-grade the channel slope in order to increase flood conveyance is particularly susceptible to such problems [E_xp_op_]. Greater flow velocities can result in more sediment from upstream riverbeds being transported and deposited in lower reaches, requiring further dredging at these sites to maintain the artificial channel form [E_xp_op_]. Removing sediment from the channel can have significant negative effects on aquatic biodiversity [D_ata_].

(25) *Bank stabilization*

River management that prevents flooding and the consequent deposit of sediment on the flood plain often results in the build-up of sediment and a reduction in the capacity of the river to move water downstream [E_xp_op_]. Conversely, where past management has destabilized banks leading to erosion and unnatural widening, bank stabilization can reduce further erosion and consequent sediment deposition and so reduce flood risk. In these cases, stabilizing banks by re-vegetation can be particular effective [E_xp_op_]. Riverbank stabilization performed to prevent *natural* bank erosion is likely to exacerbate flood risk in aggrading river reaches because, without lateral shifting, channel conveyance capacity cannot be maintained [E_xp_op_].

(26) *River restoration*

River restoration seeks to recreate natural channel properties in rivers that have been modified—often ‘channelized’—in the past. Where it increases the ability of the river to flow onto its floodplain, or creates storage in areas that were once part of the river's floodplain, river restoration can reduce flood risk downstream [E_xp_op_]. In many cases, the motive for river restoration is conservation of biodiversity, with the aim that there should be no negative flood impact.

(a) In the New Forest, river restoration has been used to reconnect channelized rivers to their floodplains. For small and medium-sized drainage basins (less than 100 km^2^) there is evidence that restoration of river channel morphology and floodplain woodland with associated large wood log-jams may reduce flood risk, although only for flows with AEP greater than 50% [D_ata_].
(b) Floodplain forest restoration can reduce peak discharge at the catchment outlet by a combination of the processes described in §§27–29. For an event with AEP of 3%, peak discharge was reduced by up to 19% under mature forest. In areas where only 20–35% of the overall catchment area was restored to forest, peak discharge was reduced by 6% [D_ata_].

(27) *Channel and bank vegetation*

Vegetation growing on banks and in the river itself can increase ‘channel roughness’ which slows water flows and increases sediment deposition. Reduced vegetation in winter reduces roughness and accounts for higher seasonal flows (by up to 50% in a study of the River Stour in Dorset) [D_ata_]. The cultivation and maintenance of bankside and river channel vegetation, which is often of value for biodiversity, can induce a small decrease in water flow and hence reduce downstream flood risk in their immediate vicinity in narrow rivers where the width is less than 16 times the depth [D_ata_].

(28) *Riparian buffer strips*

Non-agricultural riparian buffer strips of 10–30 m around channels limit catchment sediment inputs to river channels, which is important in maintaining channel conveyance [D_ata_]. Buffer strips also provide co-benefits in the form of reducing movements of agricultural pollutants into watercourses, and shading of river channels from excess heat which benefits aquatic biodiversity [D_ata_]. Wider buffer strips maintain habitat diversity and ecological functioning better than narrow ones [E_xp_op_].

(29) *Large wood*

At local scales (approx. 1 km river reaches), large items of wood caught in the channel can significantly increase the amount of water that flows over the bank, the quantity depending on the size of the items and how they are trapped (often by bridges and other man-made structures) [D_ata_]. This causes local flooding but the water stored decreases flood risk downstream [D_ata_]. During floods, wood can be mobilized and deposited at natural or artificial entrapment points in the channel. Blockage of bridges, trash racks and culverts with large wood can cause flooding upstream of the blockage [D_ata_]. Log-jams can be installed to store flood water; their effectiveness depends on log size and the density of wood entrapment sites [D_ata_].

(30) *Beavers*

In general, beaver dams reduce the mean velocity and discharge downstream of dams. Beaver ponds also trap sediment, the depth and volume of which substantially increases with dam age and frequency [D_ata_]. No evidence is available on their effects on extreme flows. Dam failures can cause minor flood waves [E_xp_op_].

(a) The effects of beaver reintroductions on flood hydrology in the UK remain to be established. The introduction of beavers in Knapdale, Argyll, Scotland, in 2009 resulted in slight changes in the configuration of woody debris in streams, although the animals in the study constructed only 0.3 dams km^−1^ (cf. 0.14–19 dams km^−1^ observed in other countries) [D_ata_], because the catchment concerned already contained well-vegetated standing water [E_xp_op_].

(31) *Summary*

The effect of modifications to river channels depends on channel cross-section, roughness and slope [D_ata_] and on where they are situated within river networks. Inappropriately located interventions may even worsen flooding due to synchronization of flood peaks. Interventions intended to reduce flooding are also likely to have effects on sedimentary, geomorphological and ecological processes in the river, as well as direct hydrological effects [E_xp_op_]. The role of sediment transport in affecting flood hazard is less well understood than that of hydraulics, but it is known that accelerated sedimentation in rivers can significantly increase downstream flood hazards, and that CBFM and NFM have the potential to reduce catchment sediment yields elevated by intensive farming [E_xp_op_].

**(e) Flood storage and floodplain conveyance**

(32) *Storage*

Water is stored naturally in catchments in forest canopies, wetlands, soils, aquifer rocks, river channels and floodplains. Management actions to increase storage may range from widespread small-scale impoundments (such as blocked ditches and micro-ponds) to large-scale flood detention reservoirs, which are major engineering works. All of these schemes store water upstream and then release it slowly over varying lengths of time depending on capacity and flood conditions. Their effectiveness at reducing flood hazard downstream depends on whether the stored water would have contributed to the flood peak. Small stores may fill up early and have no further effect in a large flood, while controllable larger storage (i.e. with gates or sluices) can be synchronized to maximize the effect on the peak of the forecast flood wave.

(a) In the 5.7 km^2^ Belford Burn catchment in north Northumberland, a pond adjacent to the river with 800–1000 m^3^ storage capacity was installed in a 0.5 km^2^ sub-catchment. During a storm in September 2008, which delivered 96 mm of rainfall in 36 h (estimated to be a rainfall event with AEP of 2%), the pond increased the average time-to-peak by 15 min [D_ata_]. Several such features would thus be necessary in order to achieve a major reduction in flood hazard (and once full cannot help if a further event occurs before they have drained) [E_xp_op_].
(b) The ‘Slowing the Flow at Pickering’ scheme, within the 69 km^2^ Pickering Beck catchment, is designed to protect the North Yorkshire town through: (i) measures in the upland landscape (tree planting, large woody debris dams, timber-built bunds, heather bale dams within moorland drains and gullies, farm woodland, riparian woodland and buffer strips), and (ii) a clay bund and engineered off-line storage (designed to protect against a flood with 4% AEP). Initial analysis of the scheme during a period of heavy rainfall in December 2015 showed a complex relationship between rainfall and river flow, and the need for more data to assess the performance of the measures, especially against the most extreme rainfall events [E_xp_op_] figure 2.

(33) *Floodplain cross-section*

River flows that just exceed the bank-full level are stored in the floodplain, while the channel remains the main mechanism for conveying water downstream. For higher flood flows, velocities on the floodplain approach that in the channel and the floodplain has an active role in conveyance. Modifications to the floodplain cross-section, typically by encroachment of built-up development protected with flood defences, will modify these floodplain functions—by reducing the natural floodplain storage and reducing the floodplain conveyance during extreme floods. Reducing the floodplain cross-section in this way will increase the water depth in the flood plain for a given flow. On the other hand, removing these obstructions increases the floodplain cross-section and reduces water depths [D_ata_]. When flood defences are overtopped or breaches occur, their effect on water levels diminishes, although they may provide additional floodplain roughness and resistance to flow [E_xp_op_]. These modifications will not only influence local water levels but will also have downstream impacts, which can be verified with a well-calibrated hydrodynamic model. In a model study of a 5 km reach of the River Cherwell, central England, construction of embankments separating the river from its floodplain (thus reducing the floodplain cross-section) increased peak flood flows downstream by 50–150% and raised water levels by up to 0.5 m [P_rojns_].

(34) *Floodplain roughness*

Riparian and floodplain forests provide ‘floodplain roughness’ which dissipates flood energy and provides resistance at times of high flow [E_xp_op_]. Removing floodplain roughness will increase flow velocities and reduce water levels locally, although this may exacerbate flooding downstream [E_xp_op_]. However, the effects of changes in floodplain roughness tend to be very small, as floodplain flow velocities tend to be low in all but the largest of floods [E_xp_op_].

(35) *Summary*

Increasing the cross-sectional area of the floodplain (by retreating flood defences or removing other obstructions from the floodplain) provides additional storage and conveyance capacity. The relative importance of storage versus conveyance depends on the flow, with the conveyance effect dominating as floodplain flows increase. The effect of storage on flooding downstream depends on whether the stored water would have contributed to the flood peak [E_xp_op_]. Increasing the floodplain cross-section will reduce flood water levels locally [E_xp_op_]. Increasing floodplain roughness (e.g. by afforestation or other obstructive vegetation) has a very small effect on flood levels, unless flow velocities on the floodplain are of the same order as the river channel, in which case increasing roughness will slow the flow [E_xp_op_]. The downstream effects of modifications to channel conveyance can be verified with hydrodynamic models [E_xp_op_].

**(f) Co-benefits**

(36) *Co-benefits*

CBFM and NFM can yield multiple co-benefits, including mitigation of diffuse pollution from agricultural land, reduced water discolouration from peatland-fed watercourses, and mitigation of soil erosion impacts on in-stream and lake ecology. The creation and restoration of terrestrial (riparian, moorland, forest) and aquatic (river, wetland) habitats and associated carbon storage may also be significant. Additional co-benefits may include aquifer recharge and retention of water upstream that can supplement water resources at times of low flow, and protection from the adverse ecological impacts of high water temperatures. These benefits can help to reduce downstream water treatment costs, sustain the productivity of agricultural soils, preserve and enhance ecosystems and biodiversity, enhance recreational value and help build the resilience of ecosystems to other stressors, including climate change. While there are many co-benefits that might arise from CBFM/NFM, to date there have been few studies that have systematically quantified these co-benefits [E_xp_op_].

**(g) Conclusion**

(37) *Conclusion I*

The hazard associated with small floods in small catchments may be significantly reduced by CBFM and NFM although the evidence does not suggest these interventions will have a major effect on the most extreme events. Large fluvial floods are caused primarily by heavy rainfall on wet, frozen or impermeable ground. It is possible that a flood will occur that is so extreme that it will overwhelm any risk management measures or flood defences, natural or otherwise. Land-use and channel form influence the severity of these floods in a fairly subtle way [E_xp_op_]. The effectiveness of NFM and CBFM varies with the severity of the event—for example, tree shelterbelts or drain blocking may offer mitigation against small floods, but are likely to be less effective during extremely intense or prolonged high rainfall [E_xp_op_]. Actions that provide small-scale local benefits have not been shown to provide significant benefits at the spatial scale of a larger catchment [S_upp_ev_]. Although a simple extrapolation would imply that many small interventions (each creating local benefits) should combine to create large benefits at large scale, this is not always the case because (i) local benefits are attenuated downstream by the channel network, and (ii) interactions among local events mean that slowing water flow in one catchment can make a flood worse further downstream when waters from several catchments meet [E_xp_op_]. Where multiple interventions have taken place it can be difficult to disentangle the effects of an individual intervention, the effect of which depends upon catchment properties (in particular size, shape, topography, geology, soils, and both hydrological and sediment connectivity) and the extent and location of the intervention within the catchment [E_xp_op_]. With the current state of scientific knowledge, it is not possible to state unequivocally whether the lack of demonstrable effect at large scale is because noticeable flood mitigation could not be achieved in a large catchment, or because a sufficiently large-scale set of interventions have not yet been implemented [E_xp_op_].

(38) *Conclusion II*

The larger the catchment and the larger the flood, the smaller is the scope for slowing the flood or storing the floodwater to reduce the flood hazard. We highlight the following main conclusions, which are summarized graphically in figure 3*a,b*: (i) Interventions that increase the ability of soils to absorb and retain water (through changes to land cover and land management) are at their most effective in smaller floods and at smaller scales. Once soils become saturated the effect is no longer noticeable; (ii) storage (from distributed micro-ponds, through natural floodplains, to large detention basins) can be effective in reducing flood risk, depending on how much storage is provided, where it is located, and how and when it is used; and (iii) increasing the cross-sectional area of floodplains by setting back flood defences that have disconnected areas of the floodplain from the river can reduce peak river flows and flood water levels.
