# Supplementary material for: A restatement of the natural science evidence concerning catchment-based ‘natural’ flood management in the UK
Source: Proc Math Phys Eng Sci. 2017 Mar 15;473(2199):20160706. doi: 10.1098/rspa.2016.0706 (PMC5378234; doi:10.1098/rspa.2016.0706)
Supplement: Annotated Bibliography [file rspa20160706supp1.pdf]

Annotated bibliography to accompany:

A restatement of the natural science evidence concerning catchment-based 'natural' flood management in the United Kingdom

Proceedings of the Royal Society A 20160706; DOI: 10.1098/rspa.2016.0706

Simon J. Dadson, Jim W. Hall, Anna Murgatroyd, Mike Acreman, Paul Bates, Keith Beven, Louise Heathwaite, Joseph Holden, Ian P. Holman, Stuart N. Lane, Enda O'Connell, Edmund Penning-Rowsell, Nick Reynard, David Sear, Colin Thorne, and Rob Wilby

Paragraph numbers correspond to those in the main document: full references are given at the end. Website URLs were accessed on 16th September 2016.

(a) Introduction and aims

(1) *Background* The complexity of flooding and flood risk management in a UK context is discussed by Sayers et al. (2002), Pattison and Lane (2012) and Thorne (2014) and, in a broader European context, by Hall et al. (2014). The process of Integrated Flood Management is introduced by WMO (2009), and a recent review of strategic flood management is given by Sayers et al. (2015a). The SEPA Natural Flood Management Handbook gives a recent, comprehensive introduction to the principles and application of NFM (SEPA, 2016). Examples of studies that have sought to draw together evidence from multiple investigations include the Foresight Project on Flood and Coastal Defence, described in Evans et al. (2004), Thorne et al. (2007), and Evans et al. (2008). The Flood Risk Management Consortium FRMRC (2004-2011) conducted research on multiple aspects of flooding, including field and model studies of land use and sediment impacts. FRMRC Fact Sheets, academic papers and technical reports may be downloaded from the FRMRC legacy website: https://web.sbe.hw.ac.uk/frmrc/.

The role of extreme events in shaping policy on flood management is scrutinized by Lane et al. (2013) and the use of risk-based decision-making to prioritize investments in reducing exposure to flooding is discussed in detail by Hall et al. (2005a) and Hall (2010). For some UK examples of groundwater flooding (in an urban context), see MacDonald et al. (2012). The effect of urbanization on flood risk in urban areas is covered by Miller et al. (2014) in a UK context. Coastal flooding and the effects of sea level changes are analysed by Dawson et al. (2005), Dawson et al. (2009), Dawson et al. (2011), and Hall et al. (2005b).

(2) *Principles* Relevant hydrological principles are covered in introductory texts (Ward and Robinson, 1999; Shaw et al., 2011; Beven, 2012). The importance of scale in hydrology is articulated well by Dooge (1986), Blöschl and Sivapalan (1995) and, in relation to the effects of land cover on flooding, Blöschl et al. (2007). The relative importance of hillslope and channel network routing in hydrograph formation has been studied extensively as a function of catchment scale (e.g., Kirkby, 1976; Beven and Wood, 1993; Robinson et al., 1995; Botter and Rinaldo, 2003; D'Odorico and Rigon, 2003). Theoretical consideration of how hydrodynamic and geomorphic dispersion affect flood wave propagation can be found in Saco and Kumar (2002), Viglione et al. (2010), and Zoccatelli et al. (2015). An example of how this translates into flood hazard is provided for the UK by Pattison et al. (2014).

(3) *Evidence* Multi-scale analyses of the effectiveness of interventions from plot to catchment scale are given by O'Connell et al. (2007) and McIntyre et al. (2013). Theoretical work on the decomposition of risk into its components: hazard, exposure and vulnerability is summarized by Rougier et al. (2013), and discussed in detail in relation to flooding by Beven and Hall (2014).

(4) *Aim* Further examples of studies in other fields in which the strength of scientific evidence is judged in order to inform policy are given by Godfray et al. (2013); Godfray et al. (2014).

(b) Meteorological drivers of flooding

(5) *Meteorological data and trends* The UK hydrometric network is described in Dixon et al. (2013). Historical trends in rainfall since 1961 are described by Jones and Conway (1997) and Osborn et al. (2000). Analysis of upland and lowland precipitation is based on Burt and Holden (2010).

(6) *River flow variability and trends* Several writers have sought to identify trends in river flow data (Mudelsee et al., 2003; Kundzewicz et al., 2005; Svensson et al., 2006). In the UK, there are several recent accounts of historical changes in runoff and river flows (Wilby et al., 2008; Hannaford and Marsh, 2008; Hannaford, 2015; Watts et al., 2015). Trends in daily maxima and 30-day maxima between 1960 and the early 2000s have been identified in some parts of the UK, notably the north and west (Hannaford and Marsh, 2008), who also comment on the continuity of these trends in earlier periods.

(7) *Flood magnitude and frequency* The review by Hannaford (2015) is particularly comprehensive. The long-term study for the Thames is by Marsh and Harvey (2012).

(8) *Flood-rich and flood-poor periods* Flood-rich and flood-poor periods during the twentieth century are identified by Wilby and Quinn (2013), Burt et al. (2015), and Foulds and Macklin (2015), in the context of prevailing weather types. The North Atlantic Oscillation (NAO) influences the pattern of wet winters and dry summers experienced in parts of the British Isles. The NAO is controlled partly by sea surface temperatures in the Atlantic Ocean, via the Atlantic Multi-decadal Oscillation (AMO). There is much recent work on the link between NAO and hydro-climatic regime in the UK (Hannaford and Marsh, 2008; Burt and Howden, 2013; Burt et al., 2015). The role of the North Atlantic Ocean in driving the pattern of anomalously wet summers in northern Europe since the 1990s is explained by Sutton and Dong (2012). Emerging evidence suggests that the North Atlantic may be affected by climate variability in the tropical Pacific (e.g., El Niño; Hoerling et al., 2001) and the stratosphere (explained by Scaife et al., 2005).

The role of atmospheric rivers in bringing anomalously wet winters is introduced by Lavers et al. (2011) who subsequently rule out any effect on summer precipitation (Champion et al., 2015). The effect of arctic sea ice on Northern Hemisphere circulation is documented by Cohen et al. (2014). The frequency of cyclonic conditions is analysed by Matthews et al. (2015). Slingo and

Palmer (2011) summarize the state of the art with respect to seasonal predictability in a UK context.

Evidence from the sedimentary record – in the form of boulder berm and debris flow deposits dated by using lichenometry – is catalogued by Macklin and Rumsby (2007) and interpreted in the context of recent UK flooding by Foulds and Macklin (2015).

(9) *Climate change projections* Several recent reviews of the hydrological impacts of climate change are global in scope (Bates et al., 2008; Trenberth, 2011). The theoretical link between warming and precipitation is discussed by Allen and Ingram (2002). The most accessible review of the current state of the art is given by Watts et al. (2015), from which estimates of future precipitation changes quoted in this paragraph are taken. Several model-based results showing the projected effects of climate change on river flows are available (Prudhomme et al., 2012; Christierson et al., 2012; Kay et al., 2014; Kundzewicz et al., 2014) along with projections of future impacts on flood risk (Sayers et al., 2015b). Changes in the intensity of summer precipitation are investigated by Kendon et al. (2014) and reviewed in their broader context by Westra et al. (2014).

(c) The effects of land cover and land management on flooding

(10) *Historical changes in land cover* The history of British forest is covered by Hoskins (1955) and Rackham (1976), and the palaeo-ecological basis for reconstructing land cover during pre-historic times is summarized by Roberts (1998). Modern data on land cover in Great Britain are from Fuller et al. (1994) and Morton et al. (2011). Land management practices are widely known to affect the magnitude and timing of runoff generation in the local vicinity (Ferrier and Jenkins, 2009).

(11) *The influence of land cover on flooding* International studies indicate a link between land cover and hydrological response (De Roo et al., 2003; Oudin et al., 2008). Nonetheless, in large UK catchments the influence of land-cover on flood peaks is hard to discern (e.g., in the Thames, Crooks and Davies, 2001). Extensive analysis of the impact of catchment properties on flooding is contained within the Flood Studies Report and the Flood Estimation Handbook (Natural Environment Research Council, 1975; Institute of Hydrology, 1999). More recent studies include the EPSRC-funded FRMRC projects and NERC-funded Flooding from Intense Rainfall projects, which are summarized in (1).

(12) *Effects of forest cover* Houghton-Carr et al. (2013a) and Houghton-Carr et al. (2013b) provide a rapid evidence assessment for the effects of forest cover on water balance. See Marc and Robinson (2007) for a comprehensive review of the Plynlimon catchment experiments. Further information is also given in Hudson and Crane (1997) and Howe et al. (1967).

(13) *Timber harvesting; forestry operations* Findings relating to timber harvesting and its effects on flood peaks are documented by Robinson and Dupeyrat (2005). Time-dependent effects of plantation forestry have been noted in many settings (e.g., Hudson and Crane, 1997; Scott and Prinsloo, 2008). Robinson et al. (2003) review the effects of forestry in several European settings. Effects of timber harvesting on stream flows through soil compaction, construction and use of forest roads, and changes in vegetation are documented by Jones and Grant (1996), while scale dependence is shown by Dung et al. (2012). Results from the Balquhidder and Coalburn experiments are described by Johnson (1995) and Robinson (1998), respectively. The effects of mature forest and simulations of peak flows at Coalburn are reported in Birkinshaw et al. (2014).

(14) *Impact of agricultural practices* Modern trends in agricultural practices are reviewed by Wheater and Evans (2009) and Burgess and Morris (2009). For comprehensive reviews of the impacts of land management on flood generation see O'Connell et al. (2004), Beven et al. (2006), O'Connell et al. (2007) and Lane et al. (2007a). The total number of sheep and lambs in England peaked in 1990 at 20.8 million and has since declined by approximately 27%; over the same period the number of cattle and calves has declined by approximately 20% to 5.4 million (DEFRA, 2016). The effects of pasture and arable management practices on soil structural degradation in local cases are documented by Holman et al. (2003). Several studies have examined the impacts of agriculture on local-scale runoff production (Heathwaite et al., 1989; Heathwaite et al., 1990; O'Connell et al., 2004) and specifically in flood generation (Boardman et al., 1994; Boardman et al., 2003).

(15) *Impact of land drainage* Comprehensive data on the effects of land drainage on river flows are compiled by Robinson (1990) and reviewed by Robinson and Rycroft (1999). A summary of the state-of-the-art and a list of studies into the effects of drainage is given by CREW (2012). The impact of agricultural drainage on historical channel network extent is documented by Ovenden and Gregory (1980). The key practitioner reference work is Buisson et al. (2008).

(16) *Soil compaction* O'Donnell (2007) used modelling to explore the possible effect of soil compaction in the Scandal Beck sub-catchment of the River Eden on downstream flood flows. The survey of soil compaction in arable and grassland plots in south-west England by Palmer and Smith (2013) shows that the effects of intensive farming depend on soil and crop type. The studies on soil compaction at Pontbren are by Marshall et al. (2009) and Marshall et al. (2014).

(17) *Upland and peatland impacts* The complexities of managing peat in uplands are reviewed by Holden et al. (2004), Holden (2005), Acreman and Holden (2013), and O'Connell et al. (2007). Specific details of runoff production mechanisms in upland areas are outlined in Evans et al. (1999), Holden and Burt (2003), and Holden (2008). The study on overland flow velocities across peat surfaces was conducted by Holden et al. (2008) and the 11.4 km^2 catchment study at Trout Beck in the North Pennines was by Grayson et al. (2010). Modelling studies highlighting the importance of surface roughness in upland peatlands include Ballard et al. (2011), Lane and Milledge (2013), and Gao et al. (2015) while experiments on spatial sensitivity to surface roughness within headwater catchments were conducted by Gao et al. (2016). The study of heather burning by Holden et al. (2015) compared five catchments with and five without patch burning, examining differences in peat properties, water tables and streamflow response to rainfall. The Exmoor Mires Project has demonstrated the benefits for ecosystem services (e.g., water and carbon storage and improvements to water quality) that can arise from the restoration of peatlands (Grand-Clement et al., 2013).

(18) *Upland ditch blocking* Ramchunder et al. (2009) investigated the effectiveness of drain blocking. Lane and Milledge (2013) quantify the effects of upland drains on downstream flood risk. The simulation study by Ballard et al. (2012) looked in detail at when drain blocking can be effective in upland peat catchments while the other modelling studies indicate the importance of surrounding peat surface roughness (Ballard et al., 2011). The Peak District peatland revegetation and gully blocking study was documented in detail by Pilkington et al. (2015).

(19) *Impacts of urbanization and Sustainable Drainage Systems (SuDS)* Urban surfaces are often assumed to be highly impervious thus converting large proportions of rainfall into surface runoff. However, empirical studies have demonstrated that the hydrological properties of urban surfaces depend on the type of surfacing and its condition with potentially complex, seasonally variable hydrological behaviour shown for some surfaces. The

hydrological properties of different urban surfaces are covered by Mansell and Rollet (2006). Kjeldsen (2009) gives some examples of current approaches to flood frequency estimation in urban areas. Urban flooding during extreme summer convective storms was typified by the Summer 2007 floods in England and Wales (Marsh and Hannaford, 2007). Several studies have evaluated the role and effectiveness of Sustainable Drainage Systems (Smith et al., 2002; Nelson et al., 2006). The definitive technical work is The SuDS Manual (Woods Ballard et al., 2015) published by the Construction Industry Research and Information Association (CIRIA; ciria.org). The multiple-benefits of restoring natural functions in urban drainage systems and watercourses as part of 'Blue-Green' approaches to urban flood risk and water management have received attention in research and practice-led consortia (http://www.bluegreencities.ac.uk/; http://bgd.org.uk/).

(20) *Catchment-scale effects of land management practices* McIntyre and Thorne (2013) give a comprehensive review of the effects of land management on flooding. The conceptual difficulties associated with the extrapolation of results from one site and scale to another are discussed by several scholars (Blöschl et al., 2007; Viglione et al., 2010; Pattison and Lane, 2012), and results from numerical simulations reinforce this apparent complexity (Dunn and Mackay, 1995; Ewen et al., 2013). O'Connell et al. (2007) reviews how local-scale changes propagate downstream, both individually and collectively, and several catchment-scale demonstrations have been documented in the literature (Marshall et al., 2009; McIntyre et al., 2012; McIntyre et al., 2013). O'Donnell (2007) has developed a modelling method for tracking impacts through the river channel network while O'Donnell et al. (2011) have used modelling to provide catchment sensitivity maps for the impacts of land management changes. There have been several recent advances in modelling methods to investigate land management impacts on flood flows at larger scales (Bulygina et al., 2011; Bulygina et al., 2012; McIntyre et al., 2013; Bulygina et al., 2013; Ewen et al., 2013).

The Hodder study is described in O'Donnell et al. (2011) and Ewen et al. (2015). Source area risk attribution is pioneered by Ewen et al. (2013). The SCaMP programme is described by McGrath and Smith (2006) and the results concerning the impacts of changes on the flood hydrograph are given by Ewen et al. (2015).

The Pontbren study is documented in Wheater et al. (2008), Marshall et al. (2009), and Marshall et al. (2014). Modelled impacts on flood peaks at the 6 km^2 catchment scale are reported by Wheater et al. (2008), Jackson et al. (2008), and McIntyre and Marshall (2010).

Results from the River Axe are reported by Climent-Soler et al. (2009) and Archer et al. (2010).

Parrott et al. (2009) discuss the way in which the effectiveness of NFM diminishes with catchment size. The role of tributary timing on flood risk is explored in a modelling study by Pattison et al. (2014). Runoff and sediment transfer are scale-dependent processes, and hence cannot be transferred directly between scales (Deasy et al., 2011; Deasy et al., 2014).

(21) *Summary* Peer-reviewed evaluations of the effectiveness of NFM and CBFM interventions are listed in Table 2. Further practitioner-led implementations of NFM and CBFM can be found in databases compiled by the Environment Agency (Barlow et al., 2014), James Hutton Institute, and the JBA Trust. (https://www.gov.uk/government/publications/working-with-natural-processes-to-reduce-flood-risk-a-research-and-development-framework; http://www.crew.ac.uk/content/natural-flood-management-database; http://naturalprocesses.jbahosting.com/).

An evidence directory compiled by the Environment Agency (SC15005 – Working with Natural Processes – evidence base & catchment/coastal laboratories), gives a synthesis of best practice drawn from twenty projects that have implemented CBFM/NFM measures in England and Wales (http://evidence.environment-agency.gov.uk/FCERM/en/Default/FCRM/Project.aspx?ProjectID=d17370c6-135f-4ceb-8ed7-23867eaa2efd&PageID=4c167c62-3bba-475d-8965-d6528b5d5f61).

Details of the Environment Agency's Catchment Flood Management Plans can be found here: https://www.gov.uk/government/collections/catchment-flood-management-plans, and the associated legislative and policy drivers are discussed by Parrott et al. (2009).

(d) Channel flow

(22) *Geomorphic processes and river channel form* Channel morphology and stability reflect the net sediment budget, which in turn reflects: (i) the net erosion and deposition observed; and (ii) the supply and connectivity of sediment from the hillslopes and upstream reaches. Effects on floodplain and channel morphology are widely discussed in the geomorphological literature (e.g., Hey, 1979; Kleinhans et al., 2013). For sediment budgets see Hooke (2003). The simulation by Lane et al. (2007b) shows the effects of flood related sedimentation on river channel form. Slater et al. (2015); Slater (2016) detect the effects of channel capacity changes by quantifying the non-stationarity in stage-discharge records. The role of fine sediments in nutrient transport is summarized by Owens and Walling (2002). The implications of UK farming intensification for sediment-related flood risks in upland and lowland basins are investigated in doctoral theses by Henshaw (2009) and Dangerfield (2013), respectively. Results of field experiments with on-farm wetlands in Cumbria and Leicestershire are reported by Ockenden et al. (2014). The comparison of sediment yields in basins dominated by improved grassland with those from traditionally-managed[1] moorland at Pontbren is by Henshaw (2009) and Thorne et al. (2011a).

(23) *Hydraulic effects of channel modification* The hydraulic effects of modifications to the geometry of channels are governed by known physical laws of steady and unsteady flow (Jain, 2001). Hall et al. (2014) review these principles in the context of changes to the flood regime in Europe.

(24) *Sedimentary effects of channel modification* Tracking and predicting river channel adjustment and the complex interrelationships between flood conveyance, sediment transport, channel morphology and adjustment to natural and artificial disturbance is a mainstay of fluvial geomorphology (Gilbert, 1880; Mackin, 1948; Hack, 1957; Schumm, 1979). Work on the effects of channel modification on channel stability is summarized by Darby and Thorne (1996). The effect of bed lowering on bank stability is considered in detail in several studies (Schumm, 1979; Thorne and Osman, 1988; Sear et al., 1995; Harvey, 2001; Hooke, 2003). A modern set of tools provided to assess the problem of dealing with sediment in flood risk management is given by Thorne et al. (2011b).

(25) *Bank stabilization* Advances in understanding river bank erosion, its natural controls and its management are covered by Lawler et al. (1997) and Midgley et al. (2012). The impacts of artificially restricting lateral channel movement on sedimentation and in-channel conveyance are explained by Raven et al. (2010). A good

[1] Moorland managed through lower stocking densities of local breeds of sheep (Welsh Mountain instead of Merino), conservation of native vegetation and no underdrainage.

practice guide to using bank stabilization as part of sustainable river and flood risk management is provided by the Scottish Environmental Protection Agency (SEPA, 2008; SEPA, 2012). The importance of vegetation in controlling bank erosion is reviewed by Solari et al. (2016).

(26) *River restoration* The type and scale of river restoration in the UK is summarized by Sear et al. (2000) and Newson et al. (2002), with updates available via the River Restoration Centre website (http://www.therrc.co.uk). Examples of the hydrological effects of river restoration are given in modelling studies (Acreman et al., 2003) and field and modelling data (Dixon et al., 2016). Local hydraulic impacts of restoration can be found in Sear et al. (2004) with reviews in Brookes and Shields (1996). The role of large wood in headwater streams in the New Forest is investigated by Dixon and Sear (2014) and the reduction in flood risk that might be achieved with floodplain forest restoration is quantified by Dixon et al. (2016).

(27) *Channel and bank vegetation* In-channel vegetation can reduce water velocity and elevate water levels significantly thus enhancing the flood hazard at that site and upstream. Early flow-resistance guides such as those of Chow (1959) and Barnes (1967) assist with the estimation of channel roughness parameters but do not properly account for composite roughness nor the time-varying roughness of vegetation subject to different depths and flow velocities (Masterman and Thorne, 1992). HR Wallingford's more advanced Conveyance Estimation System, which partitions hydrodynamic roughness between its various components, is described in McGahey et al. (2006) and reviewed comprehensively by Knight et al. (2010). Alternatives for representing vegetation effects on roughness are reviewed by Marjoribanks et al. (2014). The study in the River Stour is documented by Bates et al. (1998); the effect of vegetation on conveyance in summer floods is widely noted (Marsh and Hannaford, 2007).

(28) *Riparian buffer strips* The effects of interventions in riparian vegetation on flood conveyance are covered by Downs and Thorne (2000). The broader range of benefits conferred by riparian buffer strips is the subject of many papers in the hydrological and freshwater biological literatures, which are reviewed by Broadmeadow and Nisbet (2004). The co-benefits for sediment management are illustrated by Owens et al. (2007) and the co-benefits for biodiversity are articulated by Correll (2005).

(29) *Large wood* The general effects of wood in rivers are documented by Abbe and Montgomery (1996) and more recently by USBR (2016). As the size of wood approaches the order of the channel width, its role alters from simply providing additional resistance to fluid flow in the channel, to a role in which the debris can exert a strong structural control on channel morphology and stability. In a UK context, the reach-scale hydraulic effects are considered by Jeffries et al. (2003) and Sear et al. (2010).

(30) *Beavers* The role of beavers is reviewed by Gurnell (1998). International evidence concerning the effectiveness of beaver dams at trapping sediment is presented by Meentemeyer and Butler (1999) and Butler and Malanson (2005). Pollock et al. (2015) give practical information on the role of beavers in river restoration. Scottish Natural Heritage has published results from the Scottish Beaver Trial in a series of reports, including Perfect et al. (2015). Several additional studies of the reintroduction of beaver are underway (e.g., the Mid-Devon Beaver Trial and the River Otter Beaver Trial) but results were not available at the time of writing

(31) *Summary* No references cited.

(e) Flood storage and floodplain conveyance

(32) *Storage* A series of European studies consider the issues associated with managing offline storage (Hooijer et al., 2004; Bronstert et al., 2007; Salazar et al., 2012). Work at Belford is described by Wilkinson et al. (2010). Similar storage features are in use at other locations, e.g., Lustrum Beck, Stockton-on-Tees, although full evaluations of their performance have not been published at the time of writing. The Pickering scheme is documented by Lane et al. (2011) and Nisbet et al. (2015). A briefing note on post-event analysis following heavy rain in December 2015 is available here: Slowing the Flow Partnership Briefing: Boxing Day 2015 Flood Event http://www.forestry.gov.uk/pdf/160329_PBeck_Boxing_Day_2015_Final.pdf/$FILE/160329_PBeck_Boxing_Day_2015_Final.pdf

(33) *Floodplain cross-section* International studies have examined floodplain modification on the Mekong (Hoa et al., 2008), Po (Di Baldassarre et al., 2009), Rhine (Vorogushyn and Merz, 2013), and Danube (Blöschl et al., 2013). Work on flood risk in the Cherwell associated with disconnection from the flood plain and the potential effects of floodplain and channel restoration is presented by Acreman et al. (2003). The effect of wetlands on flood risk is discussed by Bullock and Acreman (2003).

(34) *Floodplain roughness* The effects of riparian forest on resistance to flow are documented widely in the international literature (Everitt, 1968; Swanson and Sparks, 1990; Hupp and Osterkamp, 1996; Stromberg et al., 1993). Controls on flood peak attenuation are investigated by Valentová et al. (2010) and sensitivity to floodplain roughness is presented by Hall et al. (2005b). A study that documents a case where floodplain friction has only a limited impact on flooding is reported by Aronica et al. (2002).

(35) No references cited.

(f) Co-benefits

(36) The need for further quantitative evaluation of the co-benefits associated with nature-based solutions like CBFM/NFM is articulated by Jones et al. (2012). An analysis of net benefits associated with 25 NFM schemes by Iacob et al. (2014) highlights the need to consider ecosystem co-benefits and tradeoffs on a case-by-case basis. Co-benefits associated with the influence of riparian shade on water temperature are reviewed by Orr et al. (2015).

(g) Conclusions

(37)–(38) No references cited.

References

1. Abbe TB & Montgomery DR. (1996) Large woody debris jams, channel hydraulics and habitat formation in large rivers. *Regulated Rivers Research Management* 12: 201-221.
2. Acreman M & Holden J. (2013) How wetlands affect floods. *Wetlands* 33: 773-786.
3. Acreman MC, Riddington R & Booker DJ. (2003) Hydrological impacts of floodplain restoration: A case study of the River Cherwell, UK. *Hydrology and Earth System Sciences* 7: 75-85.
4. Allen MR & Ingram WJ. (2002) Constraints on future changes in climate and the hydrological cycle. *Nature* 419: 224-232.
5. Archer DR, Climent-Soler D & Holman IP. (2010) Changes in discharge rise and fall rates applied to impact assessment of catchment land use. *Hydrology Research* 41: 13-26.
6. Aronica G, Bates PD & Horritt MS. (2002) Assessing the uncertainty in distributed model predictions using observed binary pattern information within GLUE. *Hydrological Processes* 16: 2001-2016.
7. Ballard CE, McIntyre N & Wheater HS. (2012) Effects of peatland drainage management on peak flows. *Hydrology and Earth System Sciences* 16: 2299-2310.
8. Ballard CE, McIntyre N, Wheater HS, Holden J & Wallage ZE. (2011) Hydrological modelling of drained blanket peat. *Journal of Hydrology* 407: 81-93.
9. Barlow B, Moore F & Burgess-Gamble L. (2014) *Working with natural processes to reduce flood risk,* Bristol: Environment Agency R&D framework: Science Report SC130004/R2.
10. Barnes HH. (1967) Roughness characteristics of natural channels. Washington, D.C. : U.S. Government Printing Office.
11. Bates BC, Kundzewicz ZW, Wu S & Palutikof JP. (2008) Climate Change and Water. Geneva: IPCC Secretariat.
12. Bates PD, Stewart MD, Siggers GB, Smith CN, Hervouet J-M & Sellin RHJ. (1998) Internal and external validation of a two dimensional finite element model for river flood simulation. *Proceedings of the Institution of Civil Engineers, Water Maritime and Energy* 130: 127-141.
13. Beven K. (2012) *Rainfall-Runoff Modelling: the Primer,* Chichester: Wiley.
14. Beven K & Wood EF. (1993) Flow routing and the hydrological response of channel networks. In: Beven K & Kirkby M (eds) *Channel Network Hydrology.* Chichester: Wiley, 99-128.
15. Beven K, Young P, Romanowicz R, O'Connell E, Ewen J, O'Donnell G, Hollis J, Morris J, Holman I, Lamb R, Rose S & Archer D. (2006) Project FD2120: Analysis of Historical Data Sets to look for Impacts of Land Use and Management Change on Flood Generation. Final Science Report to the Environment Agency.
16. Beven KJ & Hall JW. (2014) *Applied Uncertainty Analysis in Flood Risk Management,* London: Imperial College Press.
17. Birkinshaw SJ, Bathurst JC & Robinson M. (2014) 45 years of non-stationary hydrology over a forest plantation growth cycle, Coalburn catchment, Northern England. *Journal of Hydrology* 519, Part A: 559-573.
18. Blöschl G, Ardoin-Bardin S, Bonell M, Dorninger M, Goodrich D, Gutknecht D, Matamoros D, Merz B, Shand P & Szolgay J. (2007) At what scales do climate variability and land cover change impact on flooding and low flows? *Hydrological Processes* 21: 1241-1247.
19. Blöschl G, Nester T, Komma J, Parajka J & Perdigão RAP. (2013) The June 2013 flood in the Upper Danube Basin, and comparisons with the 2002, 1954 and 1899 floods. *Hydrology and Earth System Sciences* 17: 5197-5212.
20. Blöschl G & Sivapalan M. (1995) Scale issues in hydrological modelling: A review. *Hydrological Processes* 9: 251-290.
21. Boardman J, Evans R & Ford J. (2003) Muddy floods on the South Downs, southern England: Problem and responses. *Environmental Science and Policy* 6: 69-83.
22. Boardman J, Ligneau L, de Roo A & Vandaele K. (1994) Flooding of property by runoff from agricultural land in northwestern Europe. *Geomorphology* 10: 183-196.
23. Botter G & Rinaldo A. (2003) Scale effect on geomorphologic and kinematic dispersion. *Water Resources Research* 39: 1286.
24. Broadmeadow S & Nisbet TR. (2004) The effects of riparian forest management on the freshwater environment: a literature review of best management practice. *Hydrology and Earth System Sciences* 8: 286-305.
25. Bronstert A, Bárdossy A, Bismuth C, Buiteveld H, Disse M, Engel H, Fritsch U, Hundecha Y, Lammersen R, Niehoff D & Ritter N. (2007) Multi-scale modelling of land-use change and river training effects on floods in the Rhine basin. *River Research and Applications* 23: 1102-1125.
26. Brookes A & Shields D. (1996) *River Re-naturalisation,* Chichester: Wiley.
27. Buisson RSK, Wade PM, Cathcart RL, Hemmings SM, Manning CJ & Mayer L. (2008) *The Drainage Channel Biodiversity Manual: Integrating Wildlife and Flood Risk Management,* Peterborough: Association of Drainage Authorities and Natural England.
28. Bullock A & Acreman M. (2003) The role of wetlands in the hydrological cycle. *Hydrology and Earth System Sciences* 7: 358-389.
29. Bulygina N, Ballard C, McIntyre N, O'Donnell G & Wheater HS. (2012) Integrating different types of information into the hydrological model parameter estimation: Application to ungauged catchments and land use scenario analysis. *Water Resources Research* 48: W06519.
30. Bulygina N, McIntyre N & Wheater HS. (2011) Bayesian conditioning of a rainfall-runoff model for predicting flows in ungauged catchments and under land use changes. *Water Resources Research* 47: W02503.
31. Bulygina N, McIntyre N & Wheater HS. (2013) A comparison of rainfall-runoff modelling approaches for estimating impacts of rural land management on flood flows. *Hydrology Research* 44: 467-483.
32. Burgess PJ & Morris J. (2009) Agricultural technology and land use futures: The UK case. *Land Use Policy* 26: S222-229.
33. Burt TP & Holden J. (2010) Changing temperature and rainfall gradients in the British Uplands. *Climate Research* 45: 57-70.
34. Burt TP & Howden NJK. (2013) North Atlantic Oscillation amplifies orographic precipitation and river flow in upland Britain. *Water Resources Research* 49.
35. Burt TP, Jones PD & Howden NJK. (2015) An analysis of rainfall across the British Isles in the 1870s. *International Journal of Climatology* 35: 2934-2947.
36. Butler DR & Malanson GP. (2005) The geomorphic influence of beaver dams and failures of beaver dams. *Geomorphology* 71: 48-60.
37. Champion AJ, Allan RP & Lavers DA. (2015) Atmospheric rivers do not explain UK summer extreme rainfall. *Journal of Geophysical Research-Atmospheres* 120: 6731-6741.
38. Chow VT. (1959) *Open channel hydraulics,* Tokyo, Japan: McGraw-Hill.
39. Christierson B, Vidal J-P & Wade SD. (2012) Using UKCP09 probabilistic climate information for UK water resource planning. *Journal of Hydrology* 424-425: 48-67.
40. Climent-Soler D, Holman IP & Archer DR. (2009) Application of flow variability analysis to identify impacts of agricultural

land-use change on the River Axe, southwest England. *Hydrology Research* 40: 380-393.

41. Cohen J, Screen JA, Furtado JC, Barlow M, Whittleston D, Coumou D, Francis J, Dethloff K, Entekhabi D, Overland J & Jones J. (2014) Recent Arctic amplification and extreme mid-latitude weather. *Nature Geosciences* 7: 627-637.
42. Correll DL. (2005) Principles of planning and establishment of buffer zones. *Ecological Engineering* 24: 433-439.
43. CREW. (2012) *Natural flood management (NFM) knowledge system: Part 3 - The effect of land drainage on flood risk and farming practice,* Aberdeen: Centre of Expertise for Waters, James Hutton Institute.
44. Crooks S & Davies H. (2001) Assessment of land use change in the Thames catchment and its effect on the flood regime of the river. *Physics and Chemistry of the Earth, Part B: Hydrology, Oceans and Atmosphere* 26: 583-591.
45. D'Odorico P & Rigon R. (2003) Hillslope and channel contributions to the hydrologic response. *Water Resources Research* 39: 1113.
46. Dangerfield SF. (2013) The effects of sediment loading on morphology and flood risk in a lowland river system. http://eprints.nottingham.ac.uk/13659/1/Stephen_Dangerfield_PhD_Thesis_%28Dec_2013%29_FINAL_Sed_Morph_FR.pdf: University of Nottingham.
47. Darby SE & Thorne CR. (1996) Predicting stage-discharge curves in channels with bank vegetation. *Journal of Hydraulic Engineering* 122: 583-586.
48. Dawson RJ, Ball T, Werritty J, Werritty A, Hall JW & Roche N. (2011) Assessing the effectiveness of non-structural flood management measures in the Thames Estuary under conditions of socio-economic and environmental change. *Global Environmental Change* 21: 628-646.
49. Dawson RJ, Dickson ME, Nicholls RJ, Hall JW, Walkden MJA, Stansby PK, Mokrech M, Richards J, Zhou J, Milligan J, Jordan A, Pearson S, Rees J, Bates PD, Koukoulas S & Watkinson AR. (2009) Integrated analysis of risks of coastal flooding and cliff erosion under scenarios of long term change. *Climatic Change* 95: 249-288.
50. Dawson RJ, Hall JW, Bates PD & Nicholls RJ. (2005) Quantified analysis of the probability of flooding in the Thames Estuary under imaginable worst case sea-level rise scenarios. *International Journal of Water Resources Development* 21: 577-591.
51. De Roo A, Schmuck G, Perdigao V & Thielen J. (2003) The influence of historic land use changes and future planned land use scenarios on floods in the Oder catchment. *Physics and Chemistry of the Earth* 28: 1291-1300.
52. Deasy C, Baxendale SA, Heathwaite AL, Ridall G, Hodgkinson R & Brazier RE. (2011) Advancing understanding of runoff and sediment transfers in agricultural catchments through simultaneous observations across scales. *Earth Surface Processes and Landforms* 36: 1749-1760.
53. Deasy C, Titman A & Quinton JN. (2014) Measurement of flood peak effects as a result of soil and land management, with focus on experimental issues and scale. *Journal of Environmental Management* 132: 304-312.
54. DEFRA. (2016) *Structure of the Agricultural Industry: Annual statistics on the number of livestock in England and the UK in June and December,* London: DEFRA (https://www.gov.uk/government/statistical-data-sets/structure-of-the-livestock-industry-in-england-at-december).
55. Di Baldassarre G, Castellarin A & Brath A. (2009) Analysis of the effects of levee heightening on flood propagation: Example of the River Po, Italy. *Hydrological Sciences Journal* 54: 1007-1017.
56. Dixon H, Hannaford J & Fry M. (2013) The effective management of national hydrometric data – experiences from the United Kingdom. *Hydrological Sciences Journal* 58: 1383-1389.
57. Dixon SJ & Sear DA. (2014) The influence of geomorphology on large wood dynamics in a low gradient headwater stream. *Water Resources Research* 50: 9194-9210.
58. Dixon SJ, Sear DA, Odoni NA, Sykes T & Lane SN. (2016) The effects of river restoration on catchment scale flood risk and flood hydrology. *Earth Surface Processes and Landforms* 41: 997-1008.
59. Dooge JCI. (1986) Looking for hydrologic laws. *Water Resources Research* 22(9S): 46S–58S.
60. Downs PW & Thorne CR. (2000) Rehabilitation of a lowland river: Reconciling flood defence with habitat diversity and geomorphological sustainability. *Journal of Environmental Management* 58: 249-268.
61. Dung BX, Gomi T, Miyata S, Sidle RC, Kosugi K & Onda Y. (2012) Runoff responses to forest thinning at plot and catchment scales in a headwater catchment draining Japanese cypress forest. *Journal of Hydrology* 444-445: 51-62.
62. Dunn SM & Mackay R. (1995) Spatial variation in evapotranspiration and the influence of land use on catchment hydrology. *Journal of Hydrology* 171: 49-73.
63. Evans E, Ashley R, Hall J, Penning-Rowsell E, Saul A, Sayers P, Thorne C & Watkinson A. (2004) *Foresight: Future Flooding. Scientific Summary: Volume 1—Future Risks and Their Drivers,* London: Office of Science and Technology.
64. Evans EP, Simm JD, Thorne CR, Arnell NW, Ashley RM, Hess TM, Lane SN, Morris J, Nicholls RJ, Penning-Rowsell EC, Reynard NS, Saul AJ, Tapsell SM, Watkinson AR & Wheater HS. (2008) *An update of the Foresight Future Flooding 2004 qualitative risk analysis,* London (http://webarchive.nationalarchives.gov.uk/20100807034701/http:/archive.cabinetoffice.gov.uk/pittreview/_/media/assets/www.cabinetoffice.gov.uk/flooding_review/evidence/foresight_report pdf.pdf): Cabinet Office.
65. Evans MG, Burt TP, Holden J & Adamson JK. (1999) Runoff generation and water table fluctuations in blanket peat: Evidence from UK data spanning the dry summer of 1995. *Journal of Hydrology* 221: 141-160.
66. Everitt BL. (1968) Use of the cottonwood in an investigation of the recent history of a flood plain. *American Journal of Science* 266: 417-439.
67. Ewen J, Geris J, O'Connell E, O'Donnell G & Mayes W. (2015) Multiscale experimentation, monitoring and analysis of long-term land use changes and flood risk. *Project Report SC060092/R1.* Bristol: Environment Agency.
68. Ewen J, O'Donnell G, Bulygina N, Ballard C & O'Connell E. (2013) Towards understanding links between rural land management and the catchment flood hydrograph. *Quarterly Journal of the Royal Meteorological Society* 139: 350-357.
69. Ferrier RC & Jenkins A. (2009) Handbook of Catchment Management. *Handbook of Catchment Management*: 1-540.
70. Foulds SA & Macklin MG. (2015) A hydrogeomorphic assessment of twenty-first century floods in the UK. *Earth Surface Processes and Landforms* 41: 256-270.
71. Fuller RM, Sheail J & Barr CJ. (1994) The land of Britain, 1930-1990: A comparative study of field mapping and remote sensing techniques. *The Geographical Journal* 160: 173-184.
72. Gao J, Holden J & Kirkby MJ. (2015) A distributed TOPMODEL for modelling impacts of land-cover change on river flow in upland peatland catchments. *Hydrological Processes* 29: 2867-2879.

73. Gao J, Holden J & Kirkby MJ. (2016) The impact of land-cover change on flood peaks in peatland basins. *Water Resources Research* 52: 3477–3492.

74. Gilbert GK. (1880) *Report on the Geology of the Henry Mountains,* Washington DC: US Government Printing Office.

75. Godfray C, Blacquiere T, Field LM, Hails RS, Petrokofsky G, Potts SG, Raine NE, Vanbergen AJ & McLean AR. (2014) A restatement of the natural science evidence base concerning neonicotinoid insecticides and insect pollinators. *Proceedings of the Royal Society B: Biological Sciences* 281: 20140558.

76. Godfray HCJ, Donnelly CA, Kao RR, Macdonald DW, McDonald RA, Petrovsky G, Wood JLN, Woodroffe R, Young DB & McLean AR. (2013) A restatement of the natural science evidence base relevant to the control of bovine tuberculosis in Great Britain. *Proceedings of the Royal Society B: Biological Sciences* 280: 20131634.

77. Grand-Clement E, Anderson K, Smith D, Luscombe D, Gatis N, Ross M & Brazier RE. (2013) Evaluating ecosystem goods and services after restoration of marginal upland peatlands in South-West England. *J Appl Ecol* 50: 324-334.

78. Grayson R, Holden J & Rose R. (2010) Long-term change in storm hydrographs in response to peatland vegetation change. *Journal of Hydrology* 389: 336-343.

79. Gurnell AM. (1998) The hydrogeomorphological effects of beaver dam-building activity. *Progress in Physical Geography* 22: 167-189.

80. Hack JT. (1957) Studies of longitudinal stream profiles in Virginia and Maryland. *US Geological Survey Professional Paper* 294-B.

81. Hall J, Arheimer B, Borga M, Brázdil R, Claps P, Kiss A, Kjeldsen TR, Kriaučiūnienė J, Kundzewicz ZW, Lang M, Llasat MC, Macdonald N, McIntyre N, Mediero L, Merz B, Merz R, Molnar P, Montanari A, Neuhold C, Parajka J, Perdigão RaP, Plavcová L, Rogger M, Salinas JL, Sauquet E, Schär C, Szolgay J, Viglione A & Blöschl G. (2014) Understanding flood regime changes in Europe: a state-of-the-art assessment. *Hydrology and Earth System Sciences* 18: 2735-2772.

82. Hall JW. (2010) Editorial. *Journal of Flood Risk Management* 3: 1-2.

83. Hall JW, Sayers PB & Dawson RJ. (2005a) National-scale assessment of current and future flood risk in England and Wales. *Natural Hazards* 36: 147-164.

84. Hall JW, Tarantola S, Bates PD & Horritt MS. (2005b) Distributed sensitivity analysis of flood inundation model calibration. *Journal of Hydraulic Engineering* 131: 117-126.

85. Hannaford J. (2015) Climate-driven changes in UK river flows: A review of the evidence. *Progress in Physical Geography* 39: 29-48.

86. Hannaford J & Marsh T. (2008) High flow and flood trends in a network of undisturbed catchments in the UK. *International Journal of Climatology* 28: 1325-1338.

87. Harvey AM. (2001) Coupling between hillslopes and channels in upland fluvial systems: implications for landscape sensitivity, illustrated from the Howgill Fells, northwest England. *Catena* 42: 225-250.

88. Heathwaite AL, Burt TP & Trudgill ST. (1989) Runoff, sediment, and solute delivery in agricultural drainage basins: a scale-dependent approach. *IAHS Publication* 182: 175-190.

89. Heathwaite AL, Burt TP & Trudgill ST. (1990) Land-use controls on sediment production in a lowland catchment, south-west England. In: Boardman J, Foster IDL & Dearing JA (eds) *Soil Erosion on Agricultural Land.* Chichester: Wiley.

90. Henshaw AJ. (2009) Impacts of land use changes and land management practices on upland catchment sediment dynamics: Pontbren, mid-Wales. http://eprints.nottingham.ac.uk/10889/1/Henshaw_%282009%29.pdf University of Nottingham.

91. Hey RD. (1979) Flow resistance in gravel-bed rivers. *Journal of the Hydraulics Division-ASCE* 105: 365-379.

92. Hoa LTV, Shigeko H, Nhan NH & Cong TT. (2008) Infrastructure effects on floods in the Mekong River Delta in Vietnam. *Hydrological Processes* 22: 1359-1372.

93. Hoerling MP, Hurrell JW & Xu T. (2001) Tropical origins for recent North Atlantic climate change. *Science* 292: 90-92.

94. Holden J. (2005) Peatland hydrology and carbon cycling: why small-scale process matters. *Philosophical Transactions of the Royal Society A: Mathematical, Physical and Engineering Sciences* 363: 2891-2913.

95. Holden J. (2008) Upland hydrology. In: Bonn A, Hubacek K, Stewart A & Allott T (eds) *Drivers of change in upland environments.* London: Routledge, 113-134.

96. Holden J & Burt TP. (2003) Runoff production in blanket peat covered catchments. *Water Resources Research* 39: 1191.

97. Holden J, Chapman PJ & Labadz JC. (2004) Artificial drainage of peatlands: hydrological and hydrochemical process and wetland restoration. *Progress in Physical Geography* 28: 95-123.

98. Holden J, Kirkby MJ, Lane SN, Milledge DJ, Brookes CJ, Holden V & McDonald AT. (2008) Overland flow velocity and roughness properties in peatlands. *Water Resources Research* 44: W06415.

99. Holden J, Palmer SM, Johnston K, Wearing C, Irvine B, Parry L & Brown LE. (2015) Impact of prescribed burning on blanket peat hydrology. *Water Resources Research* 51: 6472-6484.

100. Holman IP, Hollis JM, Bramley ME & Thompson TRE. (2003) The contribution of soil structural degradation to catchment flooding: a preliminary investigation of the 2000 floods in England and Wales. *Hydrology and Earth System Sciences* 7: 754-765.

101. Hooijer A, Klijn F, Pedroli GBM & Van Os AG. (2004) Towards sustainable flood risk management in the Rhine and Meuse river basins: synopsis of the findings of IRMA-SPONGE. *River Research and Applications* 20: 343-357.

102. Hooke J. (2003) Coarse sediment connectivity in river channel systems: a conceptual framework and methodology. *Geomorphology* 56: 79-94.

103. Hoskins WG. (1955) *The Making of the English Landscape,* London: Hodder and Stoughton.

104. Houghton-Carr HA, Boorman DB & Heuser K. (2013a) Land use, climate change and water availability: Phase 2a Development of the Rapid Evidence Assessment protocol. Wallingford: Centre for Ecology and Hydrology.

105. Houghton-Carr HA, Boorman DB & Heuser K. (2013b) Land use, climate change and water availability: Phase 2a. Rapid Evidence Assessment: Results and synthesis. Wallingford: Centre for Ecology and Hydrology.

106. Howe GM, Slaymaker HO & Harding DM. (1967) Some aspects of the flood hydrology of the upper catchments of the Severn and Wye. *Transactions of the Institute of British Geographers* 41: 33-58.

107. Hudson JA & Crane SB. (1997) The impact of the growth of new plantation forestry on evaporation and streamflow in the Llanbrynmair catchments. *Hydrology and Earth System Sciences* 1: 463-475.

108. Hupp CR & Osterkamp WR. (1996) Riparian vegetation and fluvial geomorphic processes. *Geomorphology* 14: 277-295.

109. Iacob O, Rowan JS, Brown I & Ellis C. (2014) Evaluating wider benefits of natural flood management strategies: an ecosystem-based adaptation perspective. *Hydrology Research* 45: 774-787.

110. Institute of Hydrology. (1999) *Flood Estimation Handbook,* Crowmarsh Gifford: Institute of Hydrology.

111. Jackson BM, Wheater HS, McIntyre NR, Chell J, Francis OJ, Frogbrook Z, Marshall M, Reynolds B & Solloway I. (2008) The impact of upland land management on flooding: Insights from a multiscale experimental and modelling programme. *Journal of Flood Risk Management* 1: 71-80.

112. Jain SC. (2001) *Open-Channel Flow,* New York: Wiley.

113. Jeffries R, Darby SE & Sear DA. (2003) The influence of vegetation and organic debris on flood-plain sediment dynamics: Case study of a low-order stream in the New Forest, England. *Geomorphology* 51: 61-80.

114. Johnson RC. (1995) Effects of Upland Afforestation on Water Resources: The Balquhidder Experiment 1981-1991. *Institute of Hydrology Report 116 (2nd Edition).*

115. Jones HP, Hole DG & Zavaleta ES. (2012) Harnessing nature to help people adapt to climate change. *Nature Climate Change* 2: 504-509.

116. Jones JA & Grant GE. (1996) Peak flow responses to clear cutting and roads in small and large basins, western Cascades, Oregon. *Water Resources Research* 32: 959-974.

117. Jones PD & Conway D. (1997) Precipitation in the British Isles: An analysis of area-average data updated to 1995. *International Journal of Climatology* 17: 427-438.

118. Kay AL, Crooks SM, Davies HN, Prudhomme C & Reynard NS. (2014) Probabilistic impacts of climate change on flood frequency using response surfaces I: England and Wales. *Regional Environmental Change* 14: 1215-1227.

119. Kendon EJ, Roberts NM, Fowler HJ, Roberts MJ, Chan SC & Senior CA. (2014) Heavier summer downpours with climate change revealed by weather forecast resolution model. *Nature Climate Change* 4: 570-576.

120. Kirkby M. (1976) Tests of the random network model, and its application to basin hydrology. *Earth Surface Processes* 1: 197-212.

121. Kjeldsen TR. (2009) Modelling the impact of urbanisation on flood runoff volume. *Water Management* 162: 329-336.

122. Kleinhans MG, Ferguson RI, Lane SN & Hardy RJ. (2013) Splitting rivers at their seams: bifurcations and avulsion. *Earth Surface Processes and Landforms* 38: 47-61.

123. Knight DW, McGahey C, Lamb R & Samuels P. (2010) *Practical Channel Hydraulics: Roughness, Conveyance and Afflux,* Boca Raton, FL: CRC Press.

124. Kundzewicz ZW, Graczyk D, Maurer T, Pinskwar I, Radziejewski M, Svensson C & Szwed M. (2005) Trend detection in river flow series: 1. Annual maximum flow. *Hydrological Sciences Journal* 50: 797-810.

125. Kundzewicz ZW, Kanae S, Seneviratne SI, Handmer J, Nicholls N, Peduzzi P, Mechler R, Bouwer LM, Arnell N, Mach K, Muir-Wood R, Brakenridge GR, Kron W, Benito G, Honda Y, Takahashi K & Sherstyukov B. (2014) Flood risk and climate change: Global and regional perspectives. *Hydrological Sciences Journal* 59: 1-28.

126. Lane SN & Milledge DG. (2013) Impacts of upland open drains upon runoff generation: a numerical assessment of catchment-scale impacts. *Hydrological Processes* 27: 1701-1726.

127. Lane SN, Morris J, O'Connell PE & Quinn PF. (2007a) Rural land management response measures. In: Thorne CR (ed) *Future Flooding.* London: Thomas Telford, 217-319.

128. Lane SN, November V, Landström C & Whatmore S. (2013) Explaining rapid transitions in the practice of flood risk management. *Annals of the Association of American Geographers* 103: 330-342.

129. Lane SN, Odoni N, Landström C, Whatmore SJ, Ward N & Bradley S. (2011) Doing flood risk science differently: An experiment in radical scientific method. *Transactions of the Institute of British Geographers* 36: 15-36.

130. Lane SN, Tayefi V, Reid SC, Yu D & Hardy RJ. (2007b) Interactions between sediment delivery, channel change, climate change and flood risk in a temperate upland environment. *Earth Surface Processes and Landforms* 32: 429-446.

131. Lavers DA, Allan RP, Wood EF, Villarini G, Brayshaw DJ & Wade AJ. (2011) Winter floods in Britain are connected to atmospheric rivers. *Geophysical Research Letters* 38: L23803.

132. Lawler DM, Couperthwaite J, Bull LJ & Harris NM. (1997) Bank erosion events and processes in the Upper Severn basin. *Hydrology and Earth System Sciences* 1: 523-534.

133. MacDonald D, Dixon A, Newell A & Hallaways A. (2012) Groundwater flooding within an urbanised flood plain. *Journal of Flood Risk Management* 5: 68-80.

134. Mackin JH. (1948) Concept of the Graded River. *Geological Society of America Bulletin* 59: 463-512.

135. Macklin M & Rumsby B. (2007) Changing climate and extreme floods in the British uplands. *Transactions of the Institute of British Geographers* 32: 168-186.

136. Mansell M & Rollet F. (2006) Water balance and the behaviour of different paving surfaces. *Water and Environment Journal* 20: 7-10.

137. Marc V & Robinson M. (2007) The long-term water balance (1972–2004) of upland forestry and grassland at Plynlimon, mid-Wales. *Hydrological Sciences Journal* 11: 44-60.

138. Marjoribanks TI, Hardy RJ, Lane SN & Parsons DR. (2014) High-resolution numerical modelling of flow—vegetation interactions. *Journal of Hydraulic Research* 52: 775-793.

139. Marsh T & Harvey CL. (2012) The Thames flood series: a lack of trend in flood magnitude and a decline in maximum levels. *Hydrology Research* 43: 203-214.

140. Marsh TJ & Hannaford J. (2007) The Summer 2007 Floods in England and Wales – A Hydrological Appraisal. Wallingford: Centre for Ecology & Hydrology, 32.

141. Marshall MR, Ballard CE, Frogbrook ZL, Solloway I, McIntyre N, Reynolds B & Wheater HS. (2014) The impact of rural land management changes on soil hydraulic properties and runoff processes: results from experimental plots in upland UK. *Hydrological Processes* 28: 2617-2629.

142. Marshall MR, Francis OJ, Frogbrook ZL, Jackson BM, Mcintyre N, Reynolds B, Solloway I, Wheater HS & Chell J. (2009) The impact of upland land management on flooding: results from an improved pasture hillslope. *Hydrological Processes* 23: 464-475.

143. Masterman R & Thorne CR. (1992) Predicting influence of bank vegetation on channel capacity. *Journal of Hydraulic Engineering* 118: 1052-1058.

144. Matthews T, Murphy C, Wilby RL & Harrigan S. (2015) A cyclone climatology of the British Isles 1871-2012. *International Journal of Climatology* 36: 1299-1312.

145. McGahey C, Samuels PG & Knight DW. (2006) A practical approach to estimating the flow capacity of rivers – application and analysis. In: Alves E, Cardoso A, Leal J & Ferreira R (eds) *International Conference on Fluvial Hydraulics.* Lisbon, Portugal, 6-8 September 2006.

146. McGrath M & Smith M. (2006) Sustainable Catchment Management Programme (SCaMP): From hilltop to tap. *BHS 9th National Hydrology Symposium.* Durham, UK: British Hydrological Society.

147. McIntyre N, Ballard C, Bruen M, Bulygina N, Buytaert W, Cluckie I, Dunn S, Ehret U, Ewen J, Gelfan A, Hess T, Hughes D,

Jackson B, Kjeldsen T, Merz R, Park J-S, O'Connell E, O'Donnell G, Oudin L, Todini E, Wagener T & Wheater H. (2013) Modelling the hydrological impacts of rural land use change: current state of the science and future challenges. *Hydrology Research* In Press: 1-19.

148. McIntyre N, Ballard C, Bulygina N, Frogbrook Z, Cluckie I, Dangerfield S, Ewen J, Geris J, Henshaw A, Jackson B, Marshall M, Pagella T, Park J-S, Reynolds B, O'Connell E, O'Donnell G, Sinclair F, Solloway I, Thorne C & Wheater H. (2012) The potential for reducing flood risk through changes to rural land management: outcomes from the Flood Risk Management Research Consortium. *BHS Eleventh National Symposium, Hydrology for a changing world.*
149. McIntyre N & Marshall M. (2010) Identification of rural land management signals in runoff response. *Hydrological Processes* 24: 3521-3534.
150. McIntyre N & Thorne C. (2013) Land use management effects on flood flows and sediments - guidance on prediction. London: CIRIA.
151. Meentemeyer RK & Butler DR. (1999) Hydrogeomorphic effects of beaver dams in Glacier National Park, Montana. *Physical Geography* 20: 436-446.
152. Midgley TL, Fox GA & Heeren DM. (2012) Evaluation of the bank stability and toe erosion model (BSTEM) for predicting lateral retreat on composite streambanks. *Geomorphology* 145–146: 107-114.
153. Miller JD, Kim H, Kjeldsen TR, Packman J, Grebby S & Dearden R. (2014) Assessing the impact of urbanization on storm runoff in a peri-urban catchment using historical change in impervious cover. *Journal of Hydrology* 515: 59-70.
154. Morton D, Rowland C, Wood C, Meek L, Marston C, Smith G, Wadsworth R & Simpson IC. (2011) *Final Report for LCM2007 - the new UK land cover map,* Countryside Survey Technical Report No 11/07. (CEH Project Number: C03259). NERC/Centre for Ecology & Hydrology.
155. Mudelsee M, Börngen M, Tetzlaff G & Grünewald U. (2003) No upward trend in the occurrence of extreme floods in central Europe. *Nature* 425: 166-169.
156. Natural Environment Research Council. (1975) *Flood Studies Report,,* Swindon: NERC.
157. Nelson PA, Smith JA & Miller AJ. (2006) Evolution of channel morphology and hydrologic response in an urbanizing drainage basin. *Earth Surface Processes and Landforms* 31: 1063-1079.
158. Newson MD, Pitlick J & Sear DA. (2002) Running water: Fluvial geomorphology and river restoration. In: Perrow MR & Davy AJ (eds) *Handbook of Ecological Restoration.* Cambridge: Cambridge University Press, 133-152.
159. Nisbet T, Roe P, Marrington S, Thomas H, Broadmeadow S & Valatin G. (2015) Project RMP5455: Slowing the Flow at Pickering. Final Report to Forestry Commission *Final Report to Forestry Commission* London: DEFRA.
160. O'Donnell G, Ewen J & O'Connell PE. (2011) Sensitivity maps for impacts of land management on an extreme flood in the Hodder catchment, UK. *Physics and Chemistry of the Earth* 36: 630-637.
161. O'Donnell GM. (2007) Information Tracking for Flood Impact of Land Use and Management Change. Newcastle: Newcastle University.
162. O'Connell PE, Ewen J, O'Donnell G & Quinn P. (2007) Is there a link between agricultural land-use management and flooding? *Hydrology and Earth System Sciences* 11: 96-107.
163. O'Connell PEO, Beven K, Clements RO & Harris G. (2004) Review of Impacts of Rural Land Use and Management on Flood Generation. Impact Study Report. Appendix A: Review of UK data sources relating to the impacts of land use and management on flood generation. R&D Technical Report FD2114/TR.
164. Ockenden MC, Deasy C, Quinton JN, Surridge B & Stoate C. (2014) Keeping agricultural soil out of rivers: Evidence of sediment and nutrient accumulation within field wetlands in the UK. *Journal of Environmental Management* 135: 54-62.
165. Orr HG, Johnson MF, Wilby RL, Hatton-Ellis T & Broadmeadow S. (2015) What else do managers need to know about warming rivers? A United Kingdom perspective. *Wiley Interdisciplinary Reviews: Water* 2: 55-64.
166. Osborn TJ, Hulme M, Jones PD & Basnett TA. (2000) Observed trends in the daily intensity of United Kingdom precipitation. *International Journal of Climatology* 20: 347-364.
167. Oudin L, Andréassian V, Lerat J & Michel C. (2008) Has land cover a significant impact on mean annual streamflow? An international assessment using 1508 catchments. *Journal of Hydrology* 357: 303-316.
168. Ovenden JC & Gregory KJ. (1980) The permanence of stream networks in Britain. *Earth Surface Processes* 5: 47-60.
169. Owens P, Duzant J, Deeks L, Wood G, Morgan R & Collins A. (2007) Evaluation of contrasting buffer features within an agricultural landscape for reducing sediment and sediment-associated phosphorus delivery to surface waters. *Soil Use and Management* 23: 165-175.
170. Owens P & Walling DE. (2002) The phosphorus content of fluvial sediment in rural and industrialized river basins. *Water Research* 36: 685-701.
171. Palmer RC & Smith RP. (2013) Soil structural degradation in SW England and its impact on surface-water runoff generation. *Soil Use and Management* 29: 567-575.
172. Parrott A, Brooks W, Harmar O & Pygott K. (2009) Role of rural land use management in flood and coastal risk management. *Journal of Flood Risk Management* 2: 272-284.
173. Pattison I & Lane SN. (2012) The link between land-use management and fluvial flood risk: A chaotic conception? *Progress in Physical Geography* 36: 72-92.
174. Pattison I, Lane SN, Hardy RJ & Reaney SM. (2014) The role of tributary relative timing and sequencing in controlling large floods. *Water Resources Research* 50: 5444–5458.
175. Perfect C, Gilvear D, Law A & Willby N. (2015) The Scottish Beaver Trial: Fluvial geomorphology and river habitat 2008-2013, final report. *Scottish Natural Heritage Commissioned Report No. 683.*
176. Pilkington M, Walker J, Maskill R, Allott T & Evans M. (2015) *Restoration of blanket bogs; flood risk reduction and other ecosystem benefits, Final report of the Making Space for Water project: Moors for the Future Partnership, Edale; summary and 8 detailed annexes available at:* http://www.moorsforthefuture.org.uk/making-space-water-2.
177. Pollock MM, Lewallen G, Woodruff K, Jordan CE & Castro JM. (2015) The Beaver Restoration Guidebook: Working with Beaver to Restore Streams, Wetlands, and Floodplains. Portland, Oregon. Online at: http://www.fws.gov/oregonfwo/ToolsForLandowners/RiverScience/Beaver.asp: United States Fish and Wildlife Service, 189.
178. Prudhomme C, Dadson S, Morris D, Williamson J, Goodsell G, Crooks S, Boelee L, Davies H, Buys G, Lafon T & Watts G. (2012) Future Flows Climate: an ensemble of 1-km climate change projections for hydrological application in Great Britain. *Earth Systems Science Data* 4: 143-148.
179. Rackham O. (1976) *Trees and Woodlands in the British Landscape,* London: J. M. Dent.
180. Ramchunder SJ, Brown LE & Holden J. (2009) Environmental effects of drainage, drain-blocking and prescribed vegetation

burning in UK upland peatlands. *Progress in Physical Geography* 33: 49-79.

181. Raven EK, Lane SN & Bracken LJ. (2010) Understanding sediment transfer and morphological change for managing upland gravel-bed rivers. *Progress in Physical Geography* 34: 23-45.
182. Roberts N. (1998) *The Holocene: an Environmental History,* Oxford: Blackwell.
183. Robinson JS, Sivapalan M & Snell JD. (1995) On the relative roles of hillslope processes, channel routing, and network geomorphology in the hydrologic response of natural catchments. *Water Resources Research* 31: 3089-3101.
184. Robinson M. (1990) *Impact of improved land drainage on river flows,* Report 113: Institute of Hydrology
185. Robinson M. (1998) 30 years of forest hydrology changes at Coalburn: Water balance and extreme flows. *Hydrology and Earth System Sciences* 2: 233-238.
186. Robinson M, Cognard-Plancq AL, Cosandey C, David J, Durand P, Führer HW, Hall R, Hendriques MO, Marc V, McCarthy R, McDonnell M, Martin C, Nisbet T, O'Dea P, Rodgers M & Zollner A. (2003) Studies of the impact of forests on peak flows and baseflows: A European perspective. *Forest Ecology and Management* 186: 85-97.
187. Robinson M & Dupeyrat A. (2005) Effects of commercial timber harvesting on streamflow regimes in the Plynlimon catchments, mid-Wales. *Hydrological Processes* 19: 1213-1226.
188. Robinson M & Rycroft DW. (1999) The impact of drainage on streamflow. In: Skaggs W & van Schilfgaarde J (eds) *Agricultural Drainage.* Madison, Wisconsin: American Society of Agronomy, 753-786.
189. Rougier J, Sparks S & Hill LJ. (2013) *Risk and Uncertainty Assessment for Natural Hazards*: Cambridge University Press.
190. Saco PM & Kumar P. (2002) Kinematic dispersion in stream networks – 1. Coupling hydraulic and network geometry. *Water Resources Research* 38: 1244.
191. Salazar S, Francés F, Komma J, Blume T, Francke T, Bronstert A & Blöschl G. (2012) A comparative analysis of the effectiveness of flood management measures based on the concept of "retaining water in the landscape" in different European hydro-climatic regions. *Natural Hazards and Earth Systems Science* 12: 3287-3306.
192. Sayers PB, Galloway G, Penning-Rowsell E, Shen F, Wen K, Chen Y & Le Quesne T. (2015a) Strategic flood management: ten 'golden rules' to guide a sound approach. *International Journal of River Basin Management* 13: 137-151.
193. Sayers PB, Hall JW & Meadowcroft IC. (2002) Towards risk-based flood hazard management in the UK. *Civil Engineering* 150: 36-42.
194. Sayers PB, Horritt M, Penning-Rowsell E & McKenzie A. (2015b) *Climate Change Risk Assessment 2017: Projections of future flood risk in the UK,* London: Committee on Climate Change (Research undertaken by Sayers and Partners on behalf of the Committee on Climate Change).
195. Scaife AA, Knight JR, Vallis G & Folland CK. (2005) A stratospheric influence on the winter NAO and north Atlantic surface climate. *Geophysical Research Letters* 32: L187152.
196. Schumm SA. (1979) Geomorphic thresholds: The concept and its applications. *Transactions of the Institute of British Geographers* 4: 485-515.
197. Scott DF & Prinsloo FW. (2008) Longer-term effects of pine and eucalypt plantations on streamflow. *Water Resources Research* 44: W00A08.
198. Sear DA, Millington CE, Kitts DR & Jeffries R. (2010) Logjam controls on channel: floodplain interactions in wooded catchments and their role in the formation of multi-channel patterns. *Geomorphology* 116: 305-319.
199. Sear DA, Newson MD & Brookes A. (1995) Sediment-related river maintenance: The role of fluvial geomorphology. *Earth Surface Processes and Landforms* 20: 629-647.
200. Sear DA, Newson MD & Thorne CR. (2004) *Guidebook of Applied Fluvial Geomorphology,* Swindon: WRc.
201. Sear DA, Wilcock D, Robinson MR & Fisher KR. (2000) Channel modifications and impacts. In: Acreman M (ed) *The Changing Hydrology of the UK.* London: Routledge, 55-81.
202. SEPA. (2008) *Engineering in the Water Environment. Good Practice Guide: Bank Protection Rivers and Lochs. Document reference: WAT-SG-23,* Stirling: SEPA.
203. SEPA. (2012) *Bank Protection: Position Statement to support the implementation of the Water Environment (Controlled Activities) (Scotland) Regulations 2011. Document reference WAT-PS-07-02,* Stirling: SEPA.
204. SEPA. (2016) *Natural Flood Management Handbook,* Stirling: SEPA.
205. Shaw E, Beven K, Chappell NA & Lamb R. (2011) *Hydrology in Practice,* Abingdon: Spon Press.
206. Slater L. (2016) To what extent have changes in channel capacity contributed to flood hazard trends in England and Wales? *Earth Surface Processes and Landforms* 41: 1115–1128.
207. Slater LJ, Singer MB & Kirchner JW. (2015) Hydrologic versus geomorphic drivers of trends in flood hazard. *Geophysical Research Letters* 42: 370-376.
208. Slingo J & Palmer T. (2011) Uncertainty in weather and climate prediction. *Philosophical Transactions of the Royal Society A: Mathematical, Physical and Engineering Sciences* 369: 4751-4767.
209. Smith JA, Baeck ML, Morrison JE, Sturdevant-Rees P, Turner-Gillespie DF & Bates PD. (2002) the regional hydrology of extreme floods in an urbanizing drainage basin. *Journal of Hydrometeorology* 3: 267-282.
210. Solari L, Van Oorschot M, Belletti B, Hendriks D, Rinaldi M & Vargas-Luna A. (2016) Advances on modelling riparian vegetation—hydromorphology interactions. *River Research and Applications* 32: 164-178.
211. Stromberg JC, Richter BD, Patten DT & Wolden LG. (1993) Response of a Sonoran riparian forest to a 10-year return flood. *Great Basin Naturalist* 53: 118-130.
212. Sutton RT & Dong B. (2012) Atlantic Ocean influence on a shift in European climate in the 1990s. *Nature Geosciences* 5: 788-792.
213. Svensson C, Hannaford J, Kundzewicz ZW & Marsh T. (2006) Trends in river floods: why is there no clear signal? *IAHS Publication* 305: 1-8.
214. Swanson FJ & Sparks RE. (1990) Long-term ecological research and the invisible place. *BioScience* 40.
215. Thorne C. (2014) Geographies of UK flooding in 2013/4. *The Geographical Journal* 180: 297-309.
216. Thorne CR, Evans EP & Penning-Rowsell E. (2007) Future Flooding and Coastal Erosion Risks. London: Thomas Telford.
217. Thorne CR, Henshaw AJ & Dangerfield S. (2011a) *Catchment sediment dynamics and flood risk: Impacts of upland agricultural land management on catchment sediment dynamics at Pontbren, mid-Wales.,* EPSRC Grant No. EP/FP202511/1: FRMRC Fact Sheet
218. Thorne CR & Osman AM. (1988) Riverbank stability analysis: II applications. *Journal of Hydraulic Engineering* 114: 151-172.
219. Thorne CR, Wallerstein N, Soar P, Brookes A, Wishart D, Biedenharn D, Gibson S, Little Jr C, Mooney D, Watson CC, Green T, Coulthard T & Van de Wiel M. (2011b) Accounting

for sediment in flood risk management. In: Pender G & Faulkner H (eds) *Flood Risk Science and Management.* Oxford: Blackwell.

220. Trenberth KE. (2011) Changes in precipitation with climate change. *Climate Research* 47: 123-138.
221. USBR. (2016) *National Large Wood Manual: Assessment, Planning, Design, and Maintenance of Large Wood in Fluvial Ecosystems: Restoring Process, Function, and Structure,* http://www.usbr.gov/pn/: Bureau of Reclamation and U.S. Army Engineer Research and Development Center (USBR and ERDC).
222. Valentová J, Valenta P & Weyskrabová L. (2010) Assessing the retention capacity of a floodplain using a 2D numerical model. *Journal of Hydrology and Hydromechanics* 58: 221-232.
223. Viglione A, Chirico GB, Woods R & Blöschl G. (2010) Generalised synthesis of space–time variability in flood response: An analytical framework. *J. Hydrol.* 394: 198–212.
224. Vorogushyn S & Merz B. (2013) Flood trends along the Rhine: the role of river training. *Hydrology and Earth System Sciences* 17: 3871-3884.
225. Ward RC & Robinson MR. (1999) *Principles of Hydrology,* Maidenhead: McGraw-Hill.
226. Watts G, Battarbee R, Bloomfield JP, Crossman J, Durance I, Elliot J, Garner G, Hannah DM, Hess T, Jackson CR, Kay L, Kernan M, Knox J, Mackay J, Don T, Ormerod S, Rance J, Stuart ME, Wade AJ, Wade SD, Weatherhead K, Whitehead PG & Wilby RL. (2015) Climate change and water in the UK – past changes and future prospects. *Progress in Physical Geography* 39: 6-28.
227. Westra S, Fowler HJ, Evans JP, Alexander LV, Berg P, Johnson F, Kendon EJ, Lenderink G & Roberts NM. (2014) Future changes to the intensity and frequency of short-duration extreme rainfall. *Reviews of Geophysics* 52: 522-555.
228. Wheater H & Evans E. (2009) Land use, water management and future flood risk. *Land Use Policy* 26: 251-264.
229. Wheater HS, Reynolds B, McIntyre N, M.R. M, Jackson BM, Frogbrook ZL, Solloway I, Francis OJ & Chell J. (2008) *Impacts of upland land management on flood risk: multi-scale modelling methodology and results from the Pontbren experiment,* Flood Risk Management Research Consortium, Manchester: FRMRC Research Report UR16.
230. Wilby RL, Beven KJ & Reynard NS. (2008) Climate change and fluvial flood risk in the UK: more of the same? *Hydrological Processes* 22: 2511-2523.
231. Wilby RL & Quinn NW. (2013) Reconstructing multi-decadal variations in fluvial flood risk using atmospheric circulation patterns. *Journal of Hydrology* 487: 109-121.
232. Wilkinson ME, Quinn PF & Welton P. (2010) Runoff management during the September 2008 floods in the Belford catchment, Northumberland. *Journal of Flood Risk Management* 3: 285-295.
233. Wilson L, Wilson J, Holden J, Johnstone I, Armstrong A & Morris M. (2011) The impact of drain blocking on an upland blanket bog during storm and drought events, and the importance of sampling-scale. *Journal of Hydrology* 404: 198-208.
234. WMO. (2009) *Integrated Flood Management: Concept Paper,* Geneva: World Meteorological Organisation.
235. Woods Ballard B, Wilson S, Udale-Clarke H, Illman S, Scott T, Ashley R & Kellagher R. (2015) *The SuDS Manual,* London: CIRIA.
236. Zoccatelli D, Borga M, Chirico GB & Nikolopoulos EI. (2015) The relative role of hillslope and river network routing in the hydrologic response to spatially variable rainfall fields. *Journal of Hydrology* 531: 349-359.
