# Supplementary material for: A restatement of the natural science evidence concerning catchment-based ‘natural’ flood management in the UK
Source: Proc Math Phys Eng Sci. 2017 Mar 15;473(2199):20160706. doi: 10.1098/rspa.2016.0706 (PMC5378234; doi:10.1098/rspa.2016.0706)
Supplement: Table 2 [file rspa20160706supp2.pdf]

Table 2: Studies evaluating the effectiveness of CBFM/NFM techniques (After: Environment Agency, JBA Consulting)

Name of Scheme	Type of intervention	Location	Study area	Summary of effect	Sponsor / Funder	Ref
Belford	Off-channel storage	Northumberland	5.7 km^2	Runoff attenuation by off-channel storage feature resulted in increased time-to-peak during a 2% AEP rainfall event for 0.5 km^2 sub-catchment.	Environment Agency	1
New Forest LIFE3 (Blackwater)	Runoff attenuation features	Hampshire	12 km^2	Attenuation of flood peak due to added roughness and storage	Forestry Commission, Environment Agency, Natural England	2
Pontbren	Tree planting; ditch blocking	Mid Wales	12.5 km^2	Infiltration rates up to 67 times higher in woodland compared with pasture; tree shelterbelts simulated to reduce peak flows by 2-11% for a 0.6% AEP rainfall event in a 6 km^2 sub-catchment.	Flood Risk Management Consortium (FRMRC), Coed Cymru, Coed Cadw Woodland Trust	3
Pickering	Off-channel storage, and upland land management measures	North Yorkshire	69 km^2	Off-channel storage, and other woodland, moorland and farmland interventions are estimated to provide protection against a 4% AEP flood. The Pickering project has two types of intervention: NFM in the upland landscape, and a clay bund and engineered offline storage. The latter is what gives the 4% AEP flood protection but at a cost of approximately £3m.	Ryedale District Council North Yorkshire County Council; Local Flood Levy; Defra, Forestry Commission	4
Berwyn Drain Blocking	Drain blocking	Mid Wales	100 km^2	Drain blocking led to raised water tables and diminished flood peaks	RSPB, EU-LIFE-Nature	5
SCaMP Hodder	Tree planting, ditch blocking	Lancashire	260 km^2	Modelling study showing that upland restoration over would lead to only 2% reduction in flood peak	Environment Agency, United Utilities	6

AEP, Annual Exceedance Probability, the chance of a flood of this magnitude or greater occurring in any particular year; 1. Wilkinson et al. (2010); 2. http://www.newforestlife.org.uk/life3/; 3. Jackson et al. (2008); 4. Nisbet et al. (2015); 5. Wilson et al. (2011); 6. Ewen et al. (2013).
